# In Vitro Spectroscopic Investigation of Losartan and Glipizide Competitive Binding to Glycated Albumin: A Comparative Study

**DOI:** 10.3390/ijms25179698

**Published:** 2024-09-07

**Authors:** Agnieszka Szkudlarek

**Affiliations:** Department of Physical Pharmacy, Faculty of Pharmaceutical Sciences in Sosnowiec, Medical University of Silesia in Katowice, 40-055 Katowice, Poland; aszkudlarek@sum.edu.pl; Tel.: +48-32-364-1597

**Keywords:** polypharmacotherapy, spectroscopic investigation, losartan, glipizide, glycated albumin, diabetes, AGEs, drug–albumin binding, drug–drug interactions

## Abstract

Understanding the interaction between pharmaceuticals and serum proteins is crucial for optimizing therapeutic strategies, especially in patients with coexisting chronic diseases. The primary goal of this study was to assess the potential changes in binding affinity and competition between glipizide (GLP, a second-generation sulfonylurea hypoglycemic drug) and losartan (LOS, a medication commonly prescribed for hypertension, particularly for patients with concurrent diabetes) with non-glycated (HSA) and glycated (gHSA_GLC_, gHSA_FRC_) human serum albumin using multiple spectroscopic techniques (fluorescence, UV-visible absorption, and circular dichroism spectroscopy). The results indicated that FRC is a more effective glycation agent for HSA than GLC, significantly altering the albumin structure and affecting the microenvironment around critical amino acid residues, Trp-214 and Tyr. These modifications reduce the binding affinity of LOS and GLP to gHSA_GLC_ and gHSA_FRC_, compared to HSA, resulting in less stable drug–protein complexes. The study revealed that LOS and GLP interact nonspecifically with the hydrophobic regions of the albumin surface in both binary (ligand–albumin) and ternary systems (ligand–albumin–ligand_const_) and specifically saturate the binding sites within the protein molecule. Furthermore, the presence of an additional drug (GLP in the LOS–albumin complex or LOS in the GLP–albumin complex) complicates the interactions, likely leading to competitive binding or displacement of the initially bound drug in both non-glycated and glycated albumins. Analysis of the CD spectra suggests mutual interactions between GLP and LOS, underscoring the importance of closely monitoring patients co-administered these drugs, to ensure optimal therapeutic efficacy and safety.

## 1. Introduction

The escalating prevalence of chronic diseases, including hypertension and type 2 diabetes mellitus, has necessitated the widespread use of polypharmacotherapy. This therapeutic approach mandates a comprehensive understanding of drug interactions and their biochemical implications, mainly when pharmaceuticals interact with modified biological molecules such as glycated human serum albumin (gHSA). gHSA, a product of non-enzymatic glycation of human serum albumin (HSA), is prevalent in diabetic patients and has significant clinical implications, due to its altered structure, biological function, and physicochemical properties [1,2]. 

Albumin, the primary protein in human plasma, is essential for maintaining oncotic pressure and transporting various endogenous and exogenous substances. Furthermore, HSA possesses anti-inflammatory, antioxidant, and antithrombotic properties [3]. Under diabetic conditions, HSA undergoes glycation, which can markedly affect its drug-binding capabilities. Glycated albumin serves not only as a marker of short-term glycemic control but also influences the pharmacokinetics and pharmacodynamics of therapeutic agents, potentially altering their efficacy and safety profiles [4]. Understanding how glycation modifies albumin’s binding properties is essential for optimizing drug dosing and minimizing adverse effects in diabetic patients.

**Figure 1:**
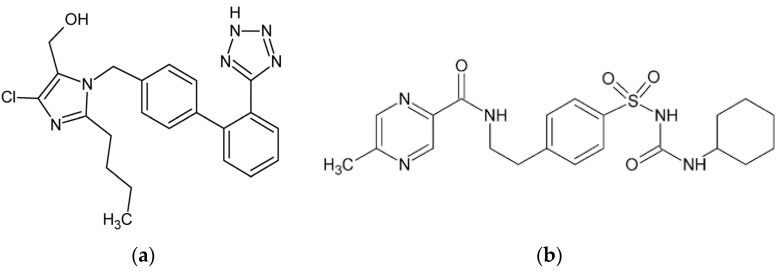
Chemical structure of (**a**) losartan (LOS) and (**b**) glipizide (GLP). The structural formulas of LOS and GLP were rendered using the ChemSketch program ver. 12.1.0.31258.

Losartan (LOS, Figure 1a) is widely used for its antihypertensive properties, primarily by inhibiting the renin–angiotensin system. By blocking angiotensin II from binding to the AT1 receptor, LOS induces vasodilation and decreases aldosterone secretion, lowering blood pressure [5,6]. LOS exhibits a high affinity to HSA, predominantly binding at site II, also known as the benzodiazepine-binding site [7]. This significant binding within subdomain IIIA of the HSA macromolecule ensures that a considerable portion of LOS remains inactive in the bloodstream, with only a minor fraction available as a free drug to exert its therapeutic effects. 

Glipizide (GLP, Figure 1b), a second-generation sulfonylurea, is widely used to manage blood glucose levels in patients with type 2 diabetes mellitus. It exerts its pharmacological effects by stimulating insulin secretion from pancreatic β-cells. This is achieved by inhibiting ATP-sensitive potassium channels on the β-cell membrane, leading to membrane depolarization and subsequent insulin release, thereby reducing blood glucose levels [8]. A study using high-performance affinity chromatography (HPAC) revealed that GLP interacts with both Sudlow’s sites I and II on HSA, but with greater affinity for site II than the site I [9]. Anwer et al. (2021) performed molecular docking and simulation studies in silico, showing that the GLP–HSA complex is formed due to hydrogen bonds and hydrophobic interactions [8].

In combination therapy, LOS and GLP may compete for the same binding site on HSA. This competitive binding can influence their pharmacokinetics and pharmacodynamics, potentially affecting their efficacy and safety profiles. The primary aim of this study was to compare the interactions of LOS and GLP with human serum albumin—both non-glycated (HSA) and glycated by glucose (gHSA_GLC_) and fructose (gHSA_FRC_)—and to investigate their mutual binding interactions using spectroscopic techniques (fluorescence spectroscopy, UV-visible absorption spectroscopy, and circular dichroism spectroscopy—CD). These techniques are widely recognized for studying in vitro interactions of drugs with albumin, due to their sensitivity, speed, and simplicity [10,11,12]. They are beneficial for calculating binding and quenching parameters, mean residue ellipticity, and characterizing proteins’ secondary and tertiary structures. 

By elucidating the competitive binding interactions of LOS and GLP with glycated albumin at a molecular level, this research seeks to provide insights that could inform better clinical practices in polypharmacotherapy, particularly for patients at risk of altered drug efficacy due to glycation. This understanding is imperative for optimizing therapeutic strategies in patients with comorbid hypertension and diabetes.

## 2. Results and Discussion

### 2.1. HSA Glycation—Formation of Glycation Products

Reducing sugars, such as glucose (GLC) and fructose (FRC), react with the primary free amino groups of the NH_2_-terminal amino acid and/or the ε-amino group of lysine (Lys), arginine (Arg), and the thiol group of cysteine (Cys-34), leading to their glycation. The final stage of this process is the formation of AGEs (advanced glycation end products), a heterogeneous group of compounds considered a critical pathophysiological factor in diabetic complications [13]. Due to a high human serum albumin (HSA) concentration, its glycation represents approximately 80% of all glycation involving circulating proteins. In healthy people, the proportion of glycated HSA is about 1–10%, while in patients with diabetes, it can increase by 2–3 times [13,14]. In vitro studies utilize various glycation agents and specific experimental conditions to initiate the glycation process. GLC, the most abundant sugar in the human body, plays a critical role in the endogenous formation of AGEs and serves as an essential biomarker in the assessment of diabetes. However, recent research has demonstrated that diabetes is also associated with abnormal fructose metabolism. Uric acid, produced from endogenous fructose, can lead to kidney and cardiovascular disease, hypertension, and obesity in diabetic patients, as FRC is generated via the polyol pathway [15,16]. Accordingly, GLC, the body’s primary energy source, and FRC, a dietary reducing sugar, were suggested to be used as glycation agents for HSA under in vitro conditions.

**Figure 2:**
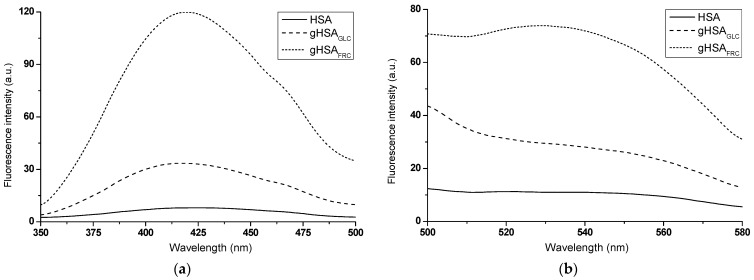
Emission fluorescence spectra of AGEs present in HSA and formed in gHSA_GLC_ and gHSA_FRC_ excited at (**a**) λ_ex_ = 335 nm and (**b**) λ_ex_ = 485 nm; protein concentration was 5 × 10^−6^ mol∙L^−1^.

Fluorescence data are often used to detect the presence of and/or monitor the kinetics of in vitro AGE formation [17]. A comparative analysis of the emission fluorescence spectra of glycation products formed in glycated albumin (gHSA_GLC_, gHSA_FRC_) and those present in non-modified HSA revealed qualitative and quantitative differences. Not only was there a significant increase in the fluorescence intensity of AGEs in the glycated proteins upon excitation at λ_ex_ = 335 nm (Figure 2a) and λ_ex_ = 485 nm (Figure 2b), but there were also notable differences in the shape of the spectra.

A widely accepted assumption is that the higher the AGE fluorescence intensity, the greater the degree of glycation [17]. From the experiment results, it is evident that FRC induced glycation of HSA more effectively than GLC. The higher fluorescence intensity of AGEs observed in gHSA_FRC_ suggests that HSA undergoes a faster and more efficient Amadori rearrangement in the presence of FRC than in the HSA–GLC system. In addition to the hyperchromic effect, this study observed a hypsochromic shift in the fluorescence maxima of glycated albumin AGEs compared to HSA excited at λ_ex_ = 335 nm (Δλ_max_ = 6 ± 1.5 nm). This blue shift indicates a decrease in polarity within the environment of the newly formed products (Figure 2a). Irradiation in the wavelength ranges of 320 nm to 335 nm and 325 nm to 335 nm caused the excitation of argpyrimidine (AP) and pentosidine (PEN), respectively. These compounds, which exhibit fluorescent properties, are formed due to protein modifications by methylglyoxal through interactions with Arg residues in macromolecules [18]. AP emits fluorescence at an approximate wavelength of λ_em_ ~ 400 nm, while PEN emits fluorescence in the range of λ_em_ = 375 nm to 385 nm [19]. The glycated albumin products gHSA_GLC_ and gHSA_FRC_, when excited at λ_ex_ = 335 nm (Figure 2a), do not display the fluorescence typical of PEN and AP. The fluorescence of AGEs at around λ_em_ = 420 nm suggests the formation of PEN and AP in glycated proteins but also indicates the presence of additional fluorophores. This is evidenced by the shift of the fluorescence maximum towards longer wavelengths compared to the emission of PEN and AP, as described by Kessel et al. (2002) [19]. An advantage of measurements at λ_ex_ = 485 nm is the absence of interference from other fluorophores in the sample. According to Schmitt et al. (2005), fluorescence with an emission maximum at λ_em_ = 530 nm (Figure 2b) appears to be an indicator of Arg residue modifications [20]. The results presented here confirmed the efficacy of the glycation process under the experimental conditions applied in this study, providing a solid foundation for further research in this area.

### 2.2. Investigating the Interaction of Losartan and Glipizide with Non-Glycated and Glycated Human Serum Albumin in Binary and Ternary Complexes

Assessing changes in the fluorescence emission intensities of aromatic amino acid residues within proteins can effectively reveal intermolecular interactions [21]. Human serum albumin (HSA) contains 18 fluorescent tyrosine residues (Tyr-9, Tyr-39, Tyr-84, Tyr-138, Tyr-140, Tyr-148, Tyr-150, Tyr-161, Tyr-263, Tyr-319, Tyr-332, Tyr-334, Tyr-340, Tyr-341, Tyr-372, Tyr-375, Tyr-401, and Tyr-411), along with a single, strongly fluorescent tryptophan residue (Trp-214) buried within a hydrophobic pocket [22]. The fluorescence quenching technique is commonly employed to determine the strength and mechanism of interactions between ligands (drugs) and albumin, especially involving the Trp-214 and Tyr residues located within albumin’s subdomains IIA and/or IB, IIB, and IIIA.

**Figure 3:**
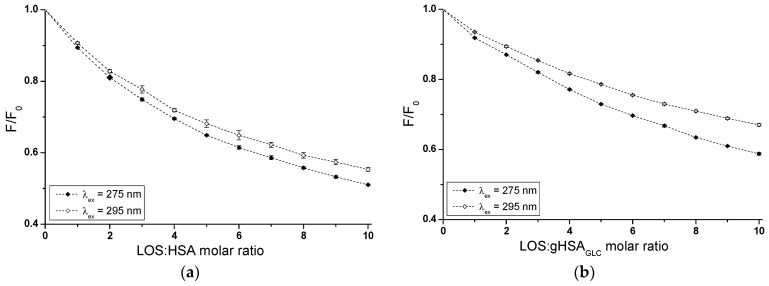

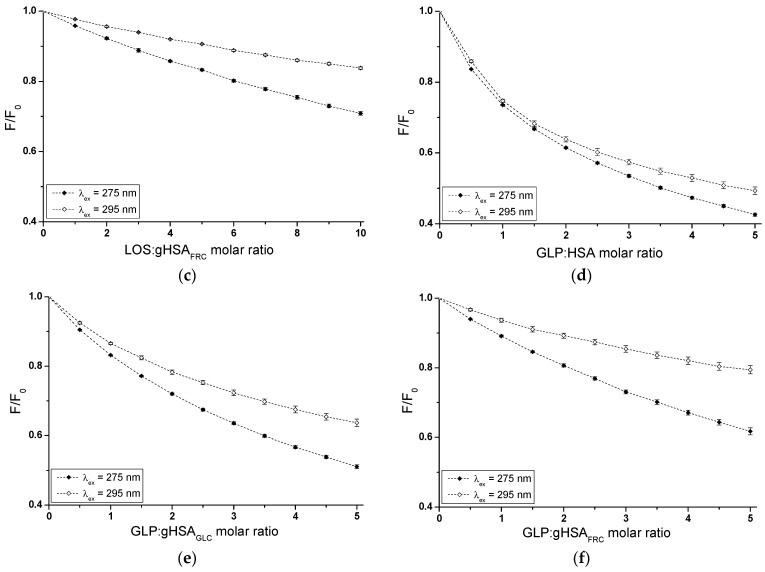
Quenching fluorescence of HSA (**a**,**d**), gHSA_GLC_ (**b**,**e**), and gHSA_FRC_ (**c**,**f**) containing 5 × 10^−6^ mol∙L^−1^ to 5 × 10^−5^ mol∙L^−1^ concentrations of LOS (**a**–**c**) and 2.5 × 10^−6^ mol∙L^−1^ to 2.5 × 10^−5^ mol∙L^−1^ concentrations of GLP (**d**–**f**). Albumin concentration: 5 × 10^−6^ mol∙L^−1^; λ_ex_ = 275 nm (◆) and λ_ex_ = 295 nm (◇).

Based on emission fluorescence spectra (Supplementary Materials Figures S1 and S2), fluorescence quenching curves of non-glycated (HSA) and glycated (gHSA_GLC_, gHSA_FRC_) albumin, excited at λ_ex_ = 275 nm and λ_ex_ = 295 nm, were plotted to determine the interaction of losartan (LOS) and glipizide (GLP) at high-affinity binding sites of albumins in the binary systems: LOS–HSA (Figure 3a), LOS–gHSA_GLC_ (Figure 3b), LOS–gHSA_FRC_ (Figure 3c), GLP–HSA (Figure 3d), GLP–gHSA_GLC_ (Figure 3e), GLP–gHSA_FRC_ (Figure 3f). 

In fluorescence studies, attention is directed towards specific protein regions containing fluorophores. Using an excitation wavelength of 275 nm enables the observation of both tryptophan (Trp-214) and tyrosine (Tyr) residues, whereas a 295 nm wavelength selectively excites Trp-214 due to its unique spectral characteristics. The primary drug-binding sites are located within the IIA and IIIA subdomains of HSA. Both hydrophobic pockets contain at least one type of the mentioned amino acids, which can transfer energy to a ligand if it is close to the fluorophore [23]. 

In Sudlow’s site I, located in the IIA subdomain, one tryptophan (Trp-214) and one tyrosine (Tyr-263) residue can participate in drug binding. Although Trp-214 is the sole tryptophan in the HSA structure, it plays a vital role in ligand interactions. The IIIA subdomain includes three tyrosine residues (Tyr-401, Tyr-411, and Tyr-497), which can transfer energy to the acceptor [24]. Additionally, Tyr-401 and Tyr-411 have been identified as amino acids that stabilize the binding of numerous ligands. To verify subdomain IIIA of human serum albumin as the specific binding site for LOS and GLP, quenching curves of HSA, gHSA_GLC_, and gHSA_FRC_ excited at λ_ex_ = 275 nm were compared with those excited at λ_ex_ = 295 nm with the addition of LOS (Figure 3a–c) and GLP (Figure 3d–f) at increasing concentrations.

As mentioned earlier, protein fluorescence quenching occurs when the distance between the chromophores in the aromatic rings of the ligand’s chemical structure and the fluorophores of albumin is less than 10 nm according to Stryer [25] and less than 7 nm according to Valeur [23]. This proximity enables fluorescence resonance energy transfer (FRET) from a donor (fluorophore) to an acceptor (chromophore), leading to non-radiative, direct energy transfer to the drug molecule. The quenching curves of HSA (Figure 3a,d), gHSA_GLC_ (Figure 3b,e), and gHSA_FRC_ (Figure 3c,f) in the presence of both LOS and GLP at increasing concentrations (with molar ratios of ligand to albumin from 1:1 to 10:1 for LOS and 1:1 to 5:1 for GLP) indicate a decrease in fluorescence for non-glycated and glycated albumin at excitation wavelengths of 275 nm and 295 nm. This indicates effective energy transfer between the albumin fluorophores and ligands. After applying corrections for the inner filter effect, the observed fluorescence quenching of HSA, gHSA_GLC_, and gHSA_FRC_ can be attributed to the formation of LOS–HSA, LOS–gHSA_GLC_, LOS–gHSA_FRC_, and GLP–HSA, GLP–gHSA_GLC_, GLP–gHSA_FRC_ complexes. 

Based on the data collected in Supplementary Materials Table S1, the percentage of non-glycated HSA fluorescence quenching, used as a control albumin, was nearly the same, reaching approximately 49.20 ± 0.32% and 45.17 ± 0.77% for LOS and 57.86 ± 0.38% and 51.46 ± 0.82% for GLP at λ_ex_ = 275 nm and λ_ex_ = 295 nm, respectively. For glycated albumin, weaker fluorescence quenching was observed in the presence of increasing ligand concentrations compared to the control sample, with the weakest quenching seen for albumin glycated by fructose (gHSA_FRC_). LOS quenched the fluorescence of gHSA_GLC_ by 41.67 ± 0.21% at λ_ex_ = 275 nm and by 33.36 ± 0.19% at λ_ex_ = 295 nm. The fluorescence of gHSA_FRC_ decreased by 29.62 ± 0.39% at 275 nm and by 16.54 ± 0.24% at 295 nm for the same molar ratio of ligand to albumin (10:1). GLP quenched the fluorescence of gHSA_GLC_ by 49.42 ± 0.45% at λ_ex_ = 275 nm and by 37.41 ± 0.68% at λ_ex_ = 295 nm, at a molar ratio GLP:albumin of 5:1. In contrast, the fluorescence of gHSA_FRC_ at λ_ex_ = 275 nm and λ_ex_ = 295 nm decreased by 38.93 ± 0.54% and 21.46 ± 0.86%, respectively. Moreover, the data presented in Table S1 indicate that both LOS and GLP had a higher affinity towards non-glycated macromolecule than towards glycated proteins (gHSA_GLC_ and gHSA_FRC_). 

The quenching curves of albumins excited at λ_ex_ = 275 nm and λ_ex_ = 295 nm in the presence of LOS (Figure 3a–c) and GLP (Figure 3d–f) at increasing drug concentrations do not overlap above the molar ratio LOS:HSA 2:1, LOS:gHSA_GLC_ 1:1, LOS:gHSA_FRC_ 1:1, and GLP:HSA 1.5:1, GLP:gHSA_GLC_ 0.5:1. The exact course of the fluorescence quenching curves indicates that there was no difference in energy transfer between the tyrosyl residues and the ligand, suggesting that, initially, only the Trp-214 residue is likely involved in the interaction with LOS and GLP. This may allow the identification of subdomain IIA as a high-affinity binding site in the albumin structure. In contrast, a different trajectory of fluorescence quenching suggests that both Trp-214 residue in subdomain IIA and Tyr residues located in hydrophobic subdomains IB, IIB, IIIA, and IIIB are involved in the interaction with LOS and GLP in the binding site environment. As shown in Figure 3, the fluorescence quenching of HSA (Figure 3a,d), gHSA_GLC_ (Figure 3b,e), and gHSA_FRC_ (Figure 3c,f) by LOS and GLP was more pronounced when excited at λ_ex_ = 275 nm compared to λ_ex_ = 295 nm. This likely indicates a significant involvement not only of Trp-214 but also of Tyr residues in the interaction between the ligands and albumins. 

The quenching curves of non-glycated and glycated albumin in the presence of LOS (Supplementary Materials Figure S1a,b) and GLP (Supplementary Materials Figure S1c,d) exhibit significant differences in their profiles. Specifically, these differences resulted from 7.53 ± 0.11% and 11.81 ± 0.58%, and 19.58 ± 0.09% and 28.63 ± 0.53% lower quenching of gHSA_GLC_ and gHSA_FRC_ by LOS relative to the LOS–HSA system at λ_ex_ = 275 nm and λ_ex_ = 295 nm, respectively. For the GLP–gHSA_GLC_ and GLP–gHSA_FRC_ systems, the differences in the quenching curve profiles amount to 8.44 ± 0.07% and 14.05 ± 0.14% for gHSA_GLC_, and 18.93 ± 0.16% and 30 ± 0.04% for gHSA_FRC_ relative to the GLP–HSA system at λ_ex_ = 275 nm and λ_ex_ = 295 nm, respectively (Table S1). Several factors may explain these observed differences. Glycation induces conformational changes in the albumin structure, modifying the binding sites and overall protein flexibility and affecting the interaction with quenchers. Additionally, glycation alters the microenvironment around crucial amino acid residues, such as Trp-214 and Tyr, influencing their accessibility to quenchers. The binding affinity of glycated albumin for LOS and GLP may also differ due to sugar moieties, which can either hinder or facilitate quencher binding [26]. Furthermore, steric hindrance from added sugar groups can reduce quenching efficiency, while new chemical interactions and potential protein aggregation further impact the quenching dynamics. These combined factors contributed to the distinct quenching behaviors observed for non-glycated and glycated albumin at different excitation wavelengths. 

**Figure 4:**
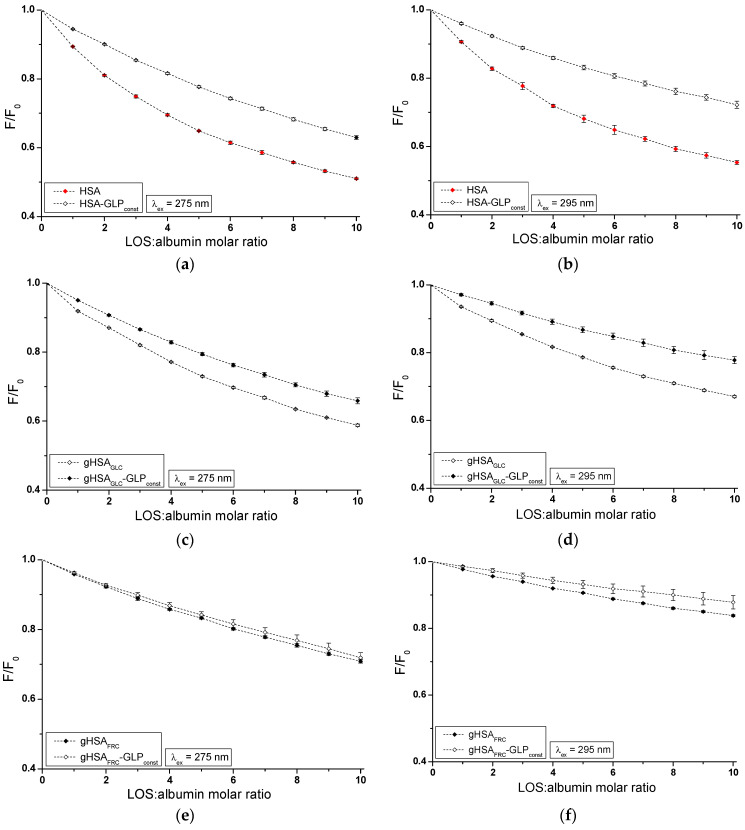
Quenching fluorescence of HSA (**a**,**b**), gHSA_GLC_ (**c**,**d**), and gHSA_FRC_ (**e**,**f**) by LOS and in the presence of GLP at 5 × 10^−6^ mol∙L^−1^ concentration. LOS concentration varied from 5 × 10^−6^ mol∙L^−1^ to 5 × 10^−5^ mol∙L^−1^. Protein concentration: 5 × 10^−6^ mol∙L^−1^; λ_ex_ = 275 nm (**a**,**c**,**e**) and λ_ex_ = 295 nm (**b**,**d**,**f**).

**Figure 5:**
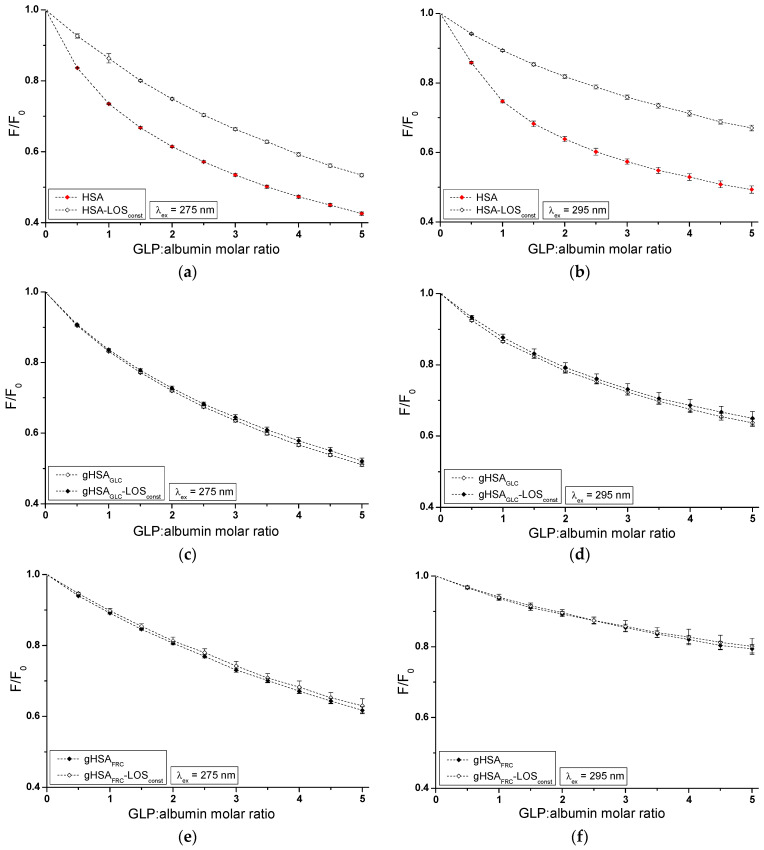
Quenching fluorescence of HSA (**a**,**b**), gHSA_GLC_ (**c**,**d**), and gHSA_FRC_ (**e**,**f**) by GLP and in the presence of LOS at 5 × 10^−6^ mol∙L^−1^ concentration. GLP concentration varied from 2.5 × 10^−6^ mol∙L^−1^ to 2.5 × 10^−5^ mol∙L^−1^. Protein concentration: 5 × 10^−6^ mol∙L^−1^; λ_ex_ = 275 nm (**a**,**c**,**e**) and λ_ex_ = 295 nm (**b**,**d**,**f**).

The influence of GLP on the LOS and LOS on the GLP affinity towards HSA, gHSA_GLC_, and gHSA_FRC_ was studied by comparing the quenching curves of albumins in the presence of LOS in the binary LOS–HSA, LOS–gHSA_GLC_, LOS–gHSA_FRC_, and ternary complexes LOS–HSA–GLP_const_, LOS–gHSA_GLC_–GLP_const_, LOS–gHSA_FRC_–GLP_const_ (Figure 4), and in the presence of GLP in the binary GLP–HSA, GLP–gHSA_GLC_, GLP–gHSA_FRC_, and ternary complexes GLP–HSA–LOS_const_, GLP–gHSA_GLC_–LOS_const_, and GLP–gHSA_FRC_–LOS_const_ (Figure 5). The Supplementary Materials (Figures S3 and S4) present emission fluorescence spectra in the binary (ligand–albumin) and ternary (ligand–albumin–ligand_const_) systems. Additionally, Figure S5 shows the quenching curves of HSA, gHSA_GLC_, and gHSA_FRC_ in the presence of LOS and GLP at λ_ex_ = 275 nm (Figure S5a,c) and at λ_ex_ = 295 nm (Figure S5b,d), respectively.

The fluorescence quenching observed in the LOS–HSA–GLP_const_ (Figure 4a,b), LOS–gHSA_GLC_–GLP_const_ (Figure 4c,d), and GLP–HSA–LOS_const_ (Figure 5a,b) systems differed from the quenching observed in the binary systems LOS–HSA (Figure 4a,b), LOS–gHSA_GLC_ (Figure 4c,d), and GLP–HSA (Figure 5a,b), respectively. The quenching of HSA and gHSA_GLC_ by LOS and HSA by GLP at maximum concentration was more pronounced by 11.64 ± 0.03% (Figure 4a), 6.64 ± 0.18% (Figure 4c), 16.69 ± 0.15% (Figure 4b), 10.04 ± 0.11% (Figure 4d), 11.76 ± 0.02% (Figure 5a), and 17.70 ± 0.21% (Figure 5b) compared to the systems with an additional ligand added to the binary system. An additional pharmaceutical in the system likely complicated the interaction between LOS–HSA and LOS–gHSA_GLC_ (or GLP–HSA) or interfered with forming these complexes. GLP (or LOS) may cause the displacement of LOS (or GLP) from its complex with non- and glycated HSA. This effect may arise from competitive binding sites on albumin, steric hindrance, or conformational alterations to the macromolecule induced by the binding of the second ligand. Furthermore, the differing affinities and binding dynamics of GLP and LOS for albumin could result in preferential binding of one ligand over the other, thereby influencing the observed quenching effect. In contrast, the absence of differences in the quenching of intrinsic fluorescence of glycated albumin by LOS or GLP in the binary systems LOS–gHSA_FRC_ (Figure 4e,f), GLP–gHSA_GLC_ (Figure 5c,d), and GLP–gHSA_FRC_ (Figure 5e,f) compared to the ternary systems LOS–gHSA_FRC_–GLP_const_ (Figure 4e,f), GLP–gHSA_GLC_–LOS_const_ (Figure 5c,d), and GLP–gHSA_FRC_–LOS_const_ (Figure 5e,f) suggests that glycation, particularly glycation of albumin by fructose, alters the macromolecule’s structure and/or binding characteristics. This modification prevents the additional drug—GLP in the LOS–gHSA_FRC_ complex or LOS in the GLP–gHSA_GLC_ and GLP–gHSA_FRC_ complexes—from competing for the binding site with LOS and GLP and prevents the displacement of drugs already bound in the gHSA_GLC_ and gHSA_FRC_ molecules. 

Additionally, the study on the LOS–HSA, LOS–gHSA_GLC_, GLP–HSA, and LOS–HSA–GLP_const_ system revealed that an increase in drug concentration leads to a hypsochromic shift (∆λ_max_) in the fluorescence emission band relative to the maximum emission of unbound albumin. This hypsochromic shift indicates an increase in the hydrophobic nature of the fluorophore environment due to drug interaction with albumin. It also suggests the possibility of hydrophobic interactions between the aromatic rings of LOS and GLP molecules and the aromatic rings of amino acid residues. The more pronounced hypsochromic shift upon excitation of albumin fluorescence at λ_ex_ = 275 nm than at λ_ex_ = 295 nm indicates a less polar environment, not only around Trp-214, but also around tyrosyl residues. The more substantial ∆λ_max_ shift towards the blue in the double systems compared to the triple systems, i.e., in the LOS–HSA system compared to LOS–HSA–GLP_const_ by 5 ± 1.5 nm, LOS–gHSA_GLC_ compared to LOS–gHSA_GLC_–GLP_const_ by 4 ± 1.5 nm, and GLP–HSA compared to GLP–HSA–GLP_const_ by 6 ± 1.5 nm, and for non-modified compared to glycated albumin, may indicate a decrease in the hydrophobic nature of the environment of tryptophan or/and tyrosyl residues of albumin after glycation and in the presence of an additional drug in the drug–albumin system.

**Figure 6:**
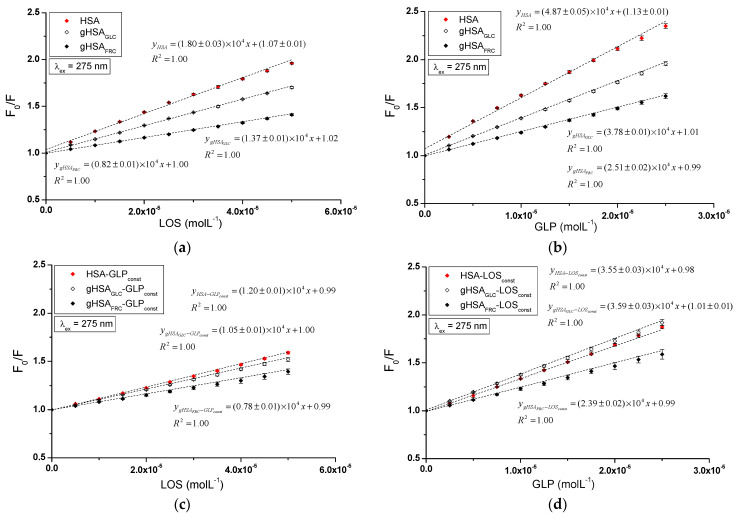
Stern–Volmer curves for the binary (**a**) LOS–HSA, LOS–gHSA_GLC_, LOS–gHSA_FRC_; (**b**) GLP–HSA, GLP–gHSA_GLC_, GLP–gHSA_FRC_, and ternary systems (**c**) LOS–HSA–GLP_const_, LOS–gHSA_GLC–_GLP_const_, LOS–gHSA_FRC_–GLP_const_; (**d**) GLP–HSA–LOS_const_, GLP–gHSA_GLC_–LOS_const_, GLP–gHSA_FRC_–LOS_const_; λ_ex_ = 275 nm.

The quenching mechanism of losartan and glipizide interaction with both non-glycated and glycated serum albumin was determined using Stern–Volmer plots. The analysis encompassed the binary systems LOS–HSA, LOS–gHSA_GLC_, LOS–gHSA_FRC_ (Figure 6a), GLP–HSA, GLP–gHSA_GLC_, GLP–gHSA_FRC_ (Figure 6b), as well as the ternary systems LOS–HSA–GLP_const_, LOS–gHSA_GLC_–GLP_const_, LOS–gHSA_FRC_–GLP_const_ (Figure 6c), GLP–HSA–LOS_const_, GLP–gHSA_GLC_–LOS_const_, GLP–gHSA_FRC_–LOS_const_ (Figure 6d) at λ_ex_ = 275 nm (Figure 6) and λ_ex_ = 295 nm (Supplementary Materials Figure S6). 

The dependence of $\frac{F_{0}}{F}$ on the LOS or GLP concentration in the binary and ternary systems at λ_ex_ = 275 nm (Figure 6) and λ_ex_ = 295 nm (Supplementary Materials Figure S6) demonstrated a linear correlation for ligand–albumin complexes. The linear Stern–Volmer plots for the ligand–albumin and ligand–albumin–ligand_const_ system may indicate a dynamic (collisional) or static quenching mechanism of fluorescence for both non-modified (HSA) and glycated albumins (gHSA_GLC_, gHSA_FRC_). According to the literature, the ligand penetrates the macromolecule’s environment in dynamic quenching, and fluorescence quenching is caused by the collision between the quencher molecule and the albumin fluorophore(s). In contrast, static quenching leads to a decrease in the intensity of the emitted fluorescence when the ligand binds to the fluorophore molecule in its ground (non-excited) state, thereby reducing the population of fluorophores capable of being excited [27,28,29]. 

Table 1 presents the Stern–Volmer constants $K_{SV}$ calculated for binary (ligand–albumin) and ternary (ligand–albumin–ligand_const_) systems at λ_ex_ = 275 nm and λ_ex_ = 295 nm. 

The magnitude of the determined fluorescence quenching rate constants $k_{q}$ (ranging from 10^12^ to 10^13^; where $k_{q}=\frac{K_{SV}}{\tau_{0}}$ and $\tau_{0}$ = 10^−9^ s) for the investigated systems indicates a static fluorescence quenching mechanism in the LOS–albumin, LOS–albumin–GLP_const_ and GLP–albumin, GLP–albumin–LOS_const_ systems (Table 1). According to Lakowicz, for collisional fluorescence quenching, the maximum value of the constant $k_{q}$ in an aqueous solution is 2 × 10^10^ (L∙mol^−1^∙s^−1^) [29]. 

The Stern–Volmer constant serves as a means to assess the accessibility of the quencher to the excited fluorophore. A higher $K_{SV}$ value indicates a greater availability of ligand molecules in the macromolecule, which leads to the formation of a complex in the excited state [30]. Glycation of HSA by glucose and fructose results in a decrease in the Stern–Volmer constant in both binary (LOS–albumin, GLP–albumin) and ternary systems (LOS–albumin–GLP_const_) at excitation wavelengths of 275 nm and 295 nm. This indicates a lower quenching efficiency of glycated albumin fluorophores compared to non-modified albumin by losartan (Table 1). In the ternary system (GLP–albumin–LOS_const_), fructose-induced glycation decreased $K_{SV}$, whereas glucose-induced glycation increased $K_{SV}$ (Table 1). The $K_{SV}$ values determined for the system in the presence of an additional drug (LOS–albumin–GLP_const_) are lower than those for the LOS–albumin system, both for unmodified and glycated albumin at λ_ex_ = 275 nm and λ_ex_ = 295 nm. The $K_{SV}$ values determined for the system in the presence of an additional drug (GLP–albumin–LOS_const_ complex) are also lower for HSA at λ_ex_ = 275 nm and λ_ex_ = 295 nm, but in contrast, for gHSA_GLC_ and gHSA_FRC_, they remain unchanged at λ_ex_ = 275 nm and λ_ex_ = 295 nm (Table 1). At both excitation wavelengths (λ_ex_ = 275 nm and λ_ex_ = 295 nm), GLP complexes exhibited higher $K_{SV}$ values than LOS complexes, indicating a greater quenching efficiency. GLP molecules are closer to fluorophores of non-modified and glycated albumin than LOS molecules in binary and ternary complexes. It is also observed that $K_{SV}$ values are generally higher at λ_ex_ = 275 nm compared to λ_ex_ = 295 nm. 

**Figure 7:**
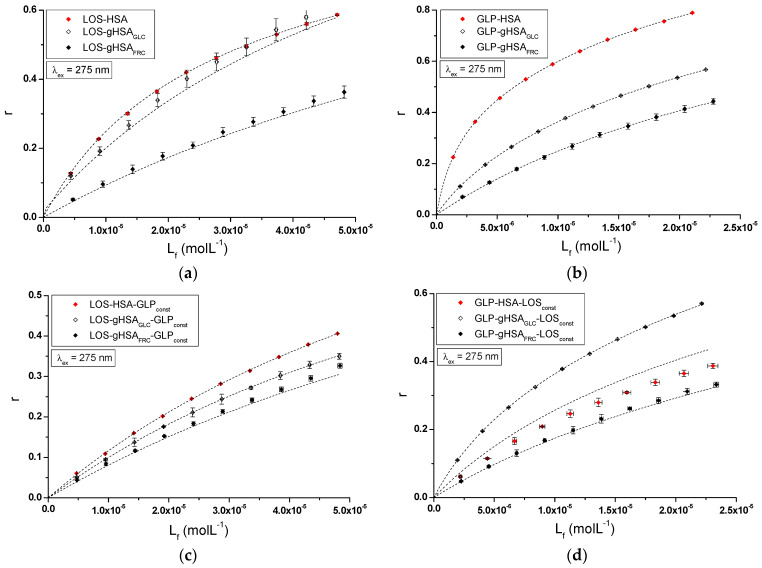
Binding isotherms of HSA, gHSA_GLC_, and gHSA_FRC_ at 5 × 10^−6^ mol∙L^−1^ concentration with LOS at 5 × 10^−6^ mol∙L^−1^ to 5 × 10^−5^ mol∙L^−1^ and GLP at 2.5 × 10^−6^ mol∙L^−1^ to 2.5 × 10^−5^ mol∙L^−1^ concentrations in the binary (**a**) LOS–HSA, LOS–gHSA_GLC_, LOS–gHSA_FRC_; (**b**) GLP–HSA, GLP–gHSA_GLC_, GLP–gHSA_FRC_ and ternary systems (**c**) LOS–HSA–GLP_const_, LOS–gHSA_GLC_–GLP_const_, LOS–gHSA_FRC_–GLP_const_; with GLP at 5 × 10^−6^ mol∙L^−1^ concentration, (**d**) GLP–HSA–LOS_const_, GLP–gHSA_GLC_–LOS_const_, GLP–gHSA_FRC_–LOS_const_ with LOS at 5 × 10^−6^ mol∙L^−1^ concentration, λ_ex_ = 275 nm.

The nature of ligand binding to albumin (specificity of binding sites within the various classes of binding sites) was determined based on the binding isotherms (saturation curves) of LOS and GLP to non-glycated and glycated HSA in binary (Figure 7a,b) and ternary systems (Figure 7c,d).

For LOS–HSA, LOS–gHSA_GLC_, LOS–gHSA_FRC_ (Figure 7a), GLP–HSA, GLP–gHSA_GLC_, GLP–gHSA_FRC_ (Figure 7b), LOS–HSA–GLP_const_, LOS–gHSA_GLC_–GLP_const_, LOS–gHSA_FRC_–GLP_const_ (Figure 7c), and GLP–HSA–LOS_const_, GLP–gHSA_GLC_–LOS_const_, GLP–gHSA_FRC_–LOS_const_ (Figure 7d) complexes at λ_ex_ = 275 nm (Figure 7) and at λ_ex_ = 295 nm (Supplementary Materials Figure S7), the course of the saturation curves is not linear across the entire range of ligand concentrations, as each of the binding isotherms exhibits an exponentially increasing course and does not reach a “plateau”. Therefore, based on the analyzed plots, it can be inferred that LOS and GLP nonspecifically interact with the hydrophobic fragments of the surface of non-glycated and glycated albumin in both binary and ternary systems and specifically saturate the binding sites within the protein molecule, as confirmed by the literature [31]. Specific binding is characterized by high affinity and low binding capacity, whereas nonspecific binding is characterized by low affinity and unlimited ligand binding capacity of the ligand [31]. The physicochemical compatibility of both molecules determines the binding of ligands to serum albumin. Small structural changes in the protein molecule can influence the mutual interaction of the drug with albumin, which in turn affects the binding parameters.

**Figure 8:**
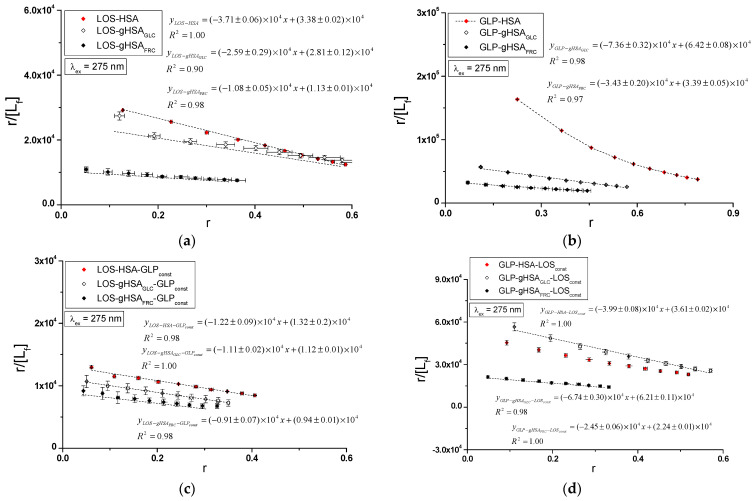
Scatchard plots for the binary (**a**) LOS–HSA, LOS–gHSA_GLC_, LOS–gHSA_FRC_; (**b**) GLP–HSA, GLP–gHSA_GLC_, GLP–gHSA_FRC_ and ternary systems (**c**) LOS–HSA–GLP_const_, LOS–gHSA_GLC_–GLP_const_, LOS–gHSA_FRC_–GLP_const_; (**d**) GLP–HSA–LOS_const_, GLP–gHSA_GLC_–LOS_const_, GLP–gHSA_FRC_–LOS_const_; λ_ex_ = 275 nm.

**Figure 9:**
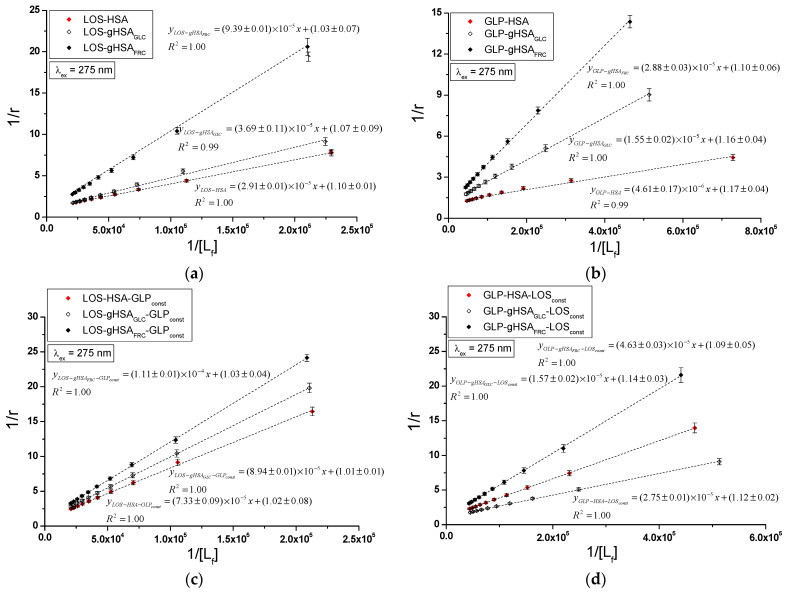
Klotz curves for the binary (**a**) LOS–HSA, LOS–gHSA_GLC_, LOS–gHSA_FRC_; (**b**) GLP–HSA, GLP–gHSA_GLC_, GLP–gHSA_FRC_ and ternary systems (**c**) LOS–HSA–GLP_const_, LOS–gHSA_GLC_–GLP_const_, LOS–gHSA_FRC_–GLP_const_; (**d**) GLP–HSA–LOS_const_, GLP–gHSA_GLC_–LOS_const_, GLP–gHSA_FRC_–LOS_const_; λ_ex_ = 275 nm.

**Figure 10:**
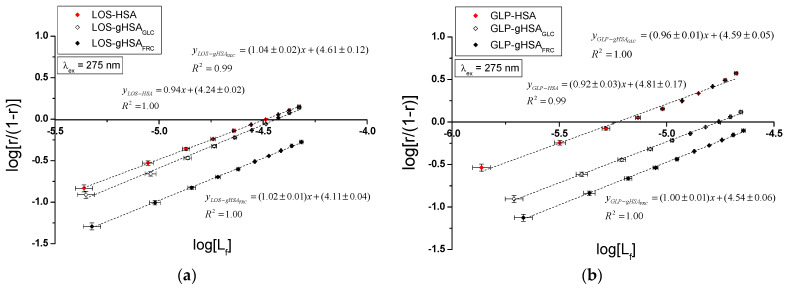

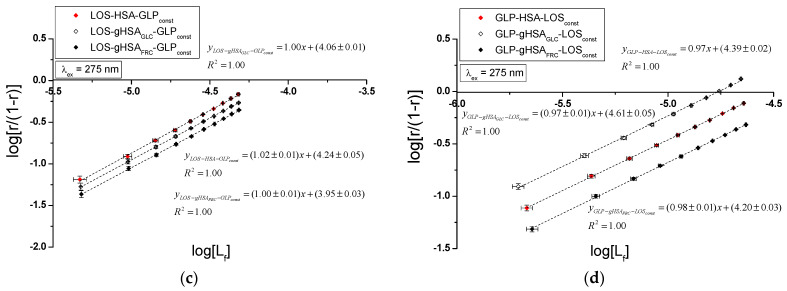
Hill plots for the binary (**a**) LOS–HSA, LOS–gHSA_GLC_, LOS–gHSA_FRC_; (**b**) GLP–HSA, GLP–gHSA_GLC_, GLP–gHSA_FRC_ and ternary systems (**c**) LOS–HSA–GLP_const_, LOS–gHSA_GLC_–GLP_const_, LOS–gHSA_FRC_–GLP_const_; (**d**) GLP–HSA–LOS_const_, GLP–gHSA_GLC_–LOS_const_, GLP–gHSA_FRC_–LOS_const_; λ_ex_ = 275 nm.

Specific binding of losartan and glipizide to non-modified and glycated albumin in the complexes LOS–HSA, LOS–gHSA_GLC_, LOS–gHSA_FRC_, GLP–HSA, GLP–gHSA_GLC_, GLP–gHSA_FRC_, LOS–HSA–GLP_const_, LOS–gHSA_GLC_–GLP_const_, LOS–gHSA_FRC_–GLP_const_ and GLP–HSA–LOS_const_, GLP–gHSA_GLC_–LOS_const_, GLP–gHSA_FRC_–LOS_const_ were quantitatively analyzed by calculating the association constant $K_{a}$ using the Scatchard equation (with the ligand-bound fraction concentration as the independent variable) (Figure 8), the Klotz equation (with the inverse of the free ligand fraction concentration as the independent variable) (Figure 9), and through non-linear regression based on the Levenberg–Marquardt algorithm, i.e., binding isotherms (Figure 7). Additionally, Hill’s coefficients $\left(n_{H}\right)$, representing cooperativity, were determined using the linear Hill plot (with the logarithm of the free ligand fraction concentration as the independent variable) (Figure 10). Changes in the high-affinity binding of LOS and GLP to non-glycated and glycated albumin in binary and ternary systems, based on $K_{a}$, the number of LOS and GLP molecules bound to one mole of the macromolecule at a specific binding site (n), as well as Hill’s coefficient of cooperativity, are summarized in Table 2 and Table 3, respectively (λ_ex_ = 275 nm, λ_ex_ = 295 nm).

The Scatchard model of ligand–protein interactions postulates that the protein molecule possesses a finite number of specific binding sites for ligands. In this case, the Scatchard dependence $\frac{r}{{[L}_{f}]}=f(r)$ is linear and intersects the x-axis of the coordinate system (the r-axis). The linear Scatchard plots for the complexes LOS–HSA, LOS–gHSA_GLC_, LOS–gHSA_FRC_ (Figure 8a), GLP–gHSA_GLC_, GLP–gHSA_FRC_ (Figure 8b), LOS–HSA–GLP_const_, LOS–gHSA_GLC_–GLP_const_, LOS–gHSA_FRC_–GLP_const_ (Figure 8c) and GLP–HSA–LOS_const_, GLP–gHSA_GLC_–LOS_const_, GLP–gHSA_FRC_–LOS_const_ (Figure 8d) at λ_ex_ = 275 nm (Figure 8) and λ_ex_ = 295 nm (Supplementary Materials Figure S8) indicate the existence of one class of equivalent, independent binding sites for LOS and GLP in both non-modified and glycated albumin structures (or a single binding site), characterized by the same association constant $K_{a}$. A non-linear Scatchard dependence resembling a hyperbola was observed for the GLP–HSA complex excited at λ_ex_ = 275 nm (Figure 8b) and λ_ex_ = 295 nm (Supplementary Materials Figure S8b). This phenomenon may have resulted from the presence of more than one class of ligand-binding sites within the albumin structure (heterogeneous binding), the non-specific nature of GLP binding to HSA, and/or negative cooperativity, where the binding of the drug at one site reduces its affinity for the remaining binding sites on the macromolecule. In contrast, for the LOS–gHSA_FRC_–GLP_const_ complex excited at λ_ex_ = 295 nm (Supplementary Materials Figure S8c), a “cone-shaped” Scatchard plot was obtained, which may indicate positive cooperativity or instability of LOS. Assuming the existence of two classes of binding sites, the binding parameters for GLP–HSA and LOS–gHSA_FRC_–GLP_const_ were determined by non-linear regression using the Levenberg–Marquardt algorithm (Table 2 and Table 3). 

Figure 9 illustrates the linear course of the Klotz dependence $\frac{1}{r}=f(\frac{1}{\left[L_{f}\right]})$ for the binary (Figure 9a,b) and ternary systems (Figure 9c,d) at λ_ex_ = 275 nm, indicating the binding of ligands to albumins within a single class of binding sites. Notably, for the system LOS–gHSA_FRC_–GLP_const_ at λ_ex_ = 295 nm, a non-linear course of the Klotz dependence was observed (Supplementary Materials Figure S9).

In the first class of binding sites, the association constants $K_{a}$ determined from the linear Scatchard and Klotz dependencies for the LOS–gHSA_GLC_ and LOS–gHSA_FRC_ complexes when excited at λ_ex_ = 275 nm and λ_ex_ = 295 nm were lower than those for the LOS–HSA complex (Table 2). A comparable trend was noted in the ternary system, where the $K_{a}$ was lower for the complexes with glycated (LOS–gHSA_GLC_–GLP_const_, LOS–gHSA_FRC_–GLP_const_) compared to non-modified albumin (LOS–HSA–GLP_const_). This suggests that albumin glycation reduced the stability of the formed complex for both the excited Trp-214 and the Tyr residues. Losartan has the lowest affinity for fructose-glycated protein (gHSA_FRC_). Moreover, the presence of an additional drug, i.e., GLP, in the LOS–albumin system weakens the binding of LOS to the macromolecules, as evidenced by a decrease in $K_{a}$ (Table 2). For the LOS–gHSA_FRC_–GLP_const_ complex excited at λ_ex_ = 295 nm, the non-linear course of the Klotz plot made it impossible to determine the binding parameters (Figure S9c).

The $K_{a}$ constants, determined by linear regression from the dependencies in the Klotz and the Scatchard equations, as well as based on binding isotherms, were significantly higher for the GLP–HSA compared to the GLP–gHSA_GLC_ and GLP–gHSA_FRC_ complexes (Table 3). This suggests that GLP has a greater affinity for non-modified than glycated albumin, forming a more stable complex. These findings align with previous studies by Koyama et al. (1997) [32], which used fluorescence quenching techniques to demonstrate that the binding capacity of hypoglycemic drugs to glycated albumin (G-HSA) was significantly lower than to non-modified HSA. Moreover, Wiglusz et al. (2021) [33] demonstrated that gliclazide, a popular hypoglycemic drug, binds more weakly to glycated albumin than its native form. These results confirm that glycation alters protein structure and drug-binding capacity. Furthermore, Chume et al. (2019) [34] confirmed that glycation of albumin decreases its binding capacity for hypoglycemic drugs, which has profound implications for the pharmacokinetics and pharmacodynamics of these drugs in diabetic patients. Conversely, for the ternary system, a higher $K_{a}$ was determined for GLP–gHSA_GLC_–LOS_const_ compared to GLP–HSA–LOS_const_ and GLP–gHSA_FRC_–LOS_const_, indicating that glucose glycation increased the stability of the formed GLP–albumin–LOS_const_ complex. Furthermore, the presence of LOS in the GLP–albumin complex generally weakened the binding of GLP to HSA and gHSA_FRC_, as reflected in a decrease in $K_{a}$ (Table 3). However, the presence of LOS did not significantly affect the $K_{a}$ value in the complex with albumin glycated by GLC at λ_ex_ = 275 nm ($K_{a}$(GLP–gHSA_GLC_) ≈ $K_{a}$(GLP–gHSA_GLC_–LOS_const_)). 

In addition, glipizide at a 5:1 GLP:albumin molar ratio had a higher affinity for non-glycated and glycated protein than losartan at a 10:1 LOS:albumin molar ratio. This effect indicates that the transfer of energy from albumin fluorophores (Trp-214 and Tyr residues) to GLP was more efficient than to LOS in both binary (ligand–albumin) and ternary (in the presence of an additional drug at a 1:1 molar ratio, ligand–albumin–ligand_const_) complexes. The number of binding sites n close to one indicates the existence of a single specific binding site for LOS and GLP in non-modified and glycated molecules (Table 2 and Table 3). 

To determine whether the binding of LOS and GLP to albumins affects the affinity of the ligand for other binding sites within the macromolecule, Hill’s coefficient $\left(n_{H}\right)$ for cooperativity was calculated based on the linear Hill plot. Figure 10 shows the linear Hill dependence $log\left(\frac{r}{1-r}\right)=f\left(log[L_{f}]\right)$ in the binary LOS–HSA, LOS–gHSA_GLC_, LOS–gHSA_FRC_ (Figure 10a), GLP–HSA, GLP–gHSA_GLC_, GLP–gHSA_FRC_ (Figure 10b), as well as the ternary systems LOS–HSA–GLP_const_, LOS–gHSA_GLC_–GLP_const_, LOS–gHSA_FRC_–GLP_const_ (Figure 10c), and GLP–HSA–LOS_const_, GLP–gHSA_GLC_–LOS_const_, GLP–gHSA_FRC_–LOS_const_ (Figure 10d) at λ_ex_ = 275 nm. Supplementary Materials, Figure S10, presents Hill plots for the binary and ternary systems at λ_ex_ = 295 nm.

For the LOS–HSA (Table 2) and GLP–HSA complexes (Table 3), the Hill coefficient values were found to be less than one ($n_{H}<1$) at both excitation wavelengths, indicating negative cooperativity. This implies that binding one LOS or GLP molecule at a binding site decreases the affinity for subsequent ligand binding at other sites on non-glycated albumin [35]. Conversely, for the LOS–gHSA_FRC_–GLP_const_ complex (Table 2), a Hill coefficient greater than one was observed ($n_{H}>1$), suggesting positive cooperativity. Here, the binding of one LOS molecule facilitated the binding affinity of additional molecules within the LOS–gHSA_FRC_–GLP_const_ complex when excited at 295 nm. For the remaining systems, the Hill coefficient was approximately one ($n_{H}$ ≈ 1), indicating non-cooperative binding of LOS and GLP to the macromolecules, where the binding of one ligand molecule does not affect the binding affinity of subsequent molecules.

### 2.3. Investigating Ligand Interactions: Spectrophotometric Analysis of Losartan and Glipizide

There are numerous possibilities for mutual interactions between different pharmaceuticals. Some of these interactions are well-documented and thus can be easily avoided during treatment. However, other medicine interactions are often uncovered only after investigating the underlying causes of treatment failure. Understanding these interactions is crucial, as it can significantly optimize therapeutic outcomes, potentially leading to better patient care and minimizing adverse effects. In this study, in addition to spectrofluorimetry, spectrophotometric measurements were conducted to verify the hypothesis that GLP and LOS can interact, not only with non-glycated (HSA) and glycated albumin (gHSA_GLC_, gHSA_FRC_) (as discussed in Section 2.2), but also with each other. 

**Figure 11:**
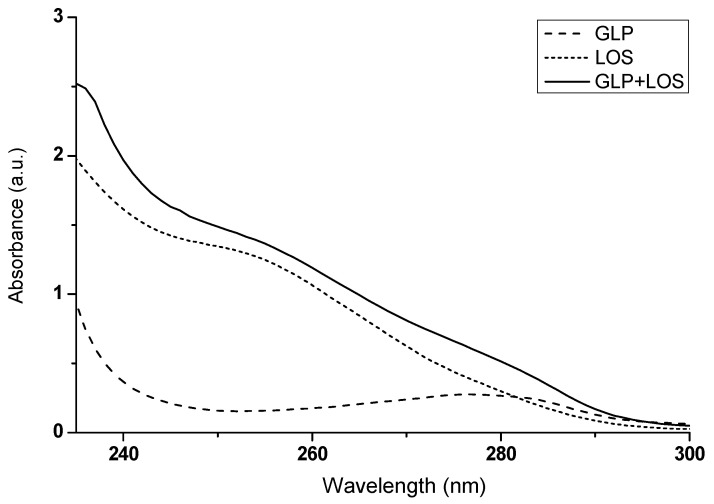
The absorption spectra of GLP, LOS, and the drug mixture (GLP + LOS); drug concentration was 2.5 × 10^−5^ mol∙L^−1^; the GLP to LOS molar ratio was 1:1; t = 37 °C.

Figure 11 shows the absorption spectra of GLP, LOS, and the drug complex (GLP + LOS), from which the absorbance values at selected wavelengths were read. The results are presented in Table 4.

If the absorbance of a mixture of two substances deviates from the mathematical sum of their individual absorbances, this may indicate potential mutual interactions, as suggested by Ren et al. (2019) [36]. By observing differences in the absorbance values of GLP and LOS in their combined mixture (GLP + LOS), compared to the expected mathematical sum of their absorbances (Figure 11, Table 4), it can be inferred that GLP and LOS interact with each other. These interactions emphasize the importance of closely monitoring patients co-administered LOS and GLP, mainly focusing on blood glucose levels, blood pressure, and renal function, to ensure safe and effective therapy.

### 2.4. Spectropolarimetric Analysis of Glycation and Losartan Influence on Macromolecule Secondary Structure 

**Figure 12:**
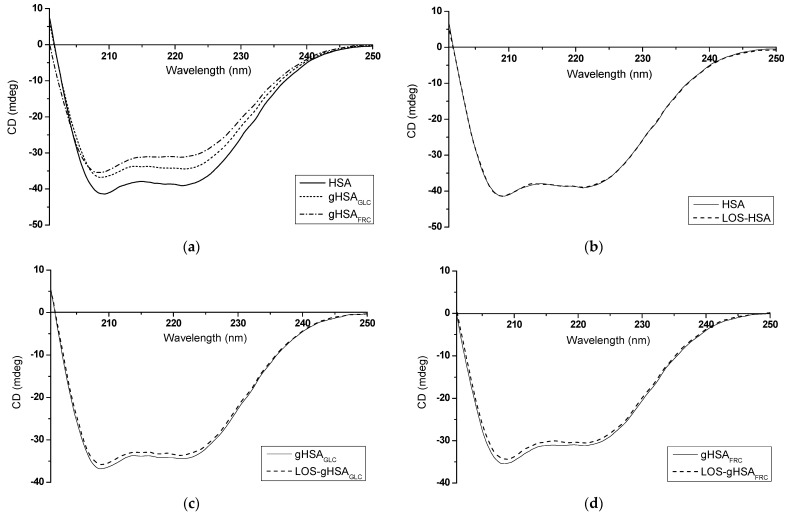
Far-UV CD spectra of (**a**) non-glycated (HSA) and glycated (gHSA_GLC_, gHSA_FRC_) human serum albumin; (**b**) HSA and LOS–HSA; (**c**) gHSA_GLC_ and LOS–gHSA_GLC_; (**d**) gHSA_FRC_ and LOS–gHSA_FRC_; protein concentration equaled 5 × 10^−6^ mol∙L^−1^; the LOS to albumin molar ratio was 10:1; t = 37 °C.

Circular dichroism (CD) spectroscopy was used to evaluate the impact of the glycation process and the presence of losartan (LOS) on the secondary structure of human serum albumin (Figure 12). CD spectroscopy is a valuable analytical technique for assessing the secondary structure of chiral molecules, particularly proteins. It can monitor conformational protein changes, such as folding/unfolding, ligand binding, and protein–protein interactions [37]. CD measurements in the far-UV region can provide quantitative assessments of secondary structure, which can be compared to findings from X-ray crystallography or NMR studies [38]. 

Table 5 and Table 6 present the value of non-glycated and glycated albumin mean residue ellipticity ${\left[\theta \right]}_{mrw}$ and the percentage content (%) of albumin secondary structure elements in the sample without (HSA, gHSA_GLC_, gHSA_FRC_) and in the presence of losartan at molar ratio of LOS:albumin 10:1 (LOS–HSA, LOS–gHSA_GLC_, LOS–gHSA_FRC_).

According to the data presented in Table 5 and Table 6, it can be inferred that both non-glycated and glycated albumin primarily display an α-helical structure. The CD spectrum of HSA is characterized by a strong double minimum at λ_min_ = 209.2 nm and λ_min_ = 221.2 nm, which shifts slightly towards shorter wavelengths due to albumin glycation (Figure 12a, Table 5). Absorption in the region below λ = 240 nm is primarily attributed to the peptide bond, featuring a weak but broad n → π* transition centered around λ = 220 nm and a more intense π → π* transition near λ = 190 nm [38]. The reduction in mean residue ellipticity ${\left[\theta \right]}_{mrw}$ and CD band intensity observed, especially in glycated gHSA_FRC_ compared to non-glycated HSA (Figure 12a, Table 5), may indicate a decrease in α-helix content (gHSA_FRC_ 16.65 ± 0.16%) and an increase in the β-sheet content of albumin after glycation by fructose (gHSA_FRC_ 6.55 ± 0.21%) (Table 6). Mou et al. (2022) observed significant changes in the secondary structure of bovine serum albumin (BSA) after glycation by ribose (RBSA). They indicated that an increase in β-sheet content in RBSA is pivotal for protein aggregation [39]. On the other hand, some authors have proposed that glycation may induce aggregation, not by unfolding, but through overall stabilization of the macromolecule, enhancing protein stability and prolonging its lifespan [40].

The formation of LOS–albumin complexes did not substantially impact the wavelength at which the spectrum reached its minimum or the mean residue ellipticity values (Table 5). As demonstrated in Table 6, the binding of LOS to HSA and gHSA_GLC_ did not lead to significant alterations in the macromolecule’s secondary structure beyond 2%. Similarly, as in this work, Żurawska-Płaksej et al. (2018) observed only slight changes in the secondary structure of non-glycated and glycated BSA upon binding with gliclazide (GLICL). The authors emphasized that the decrease in α-helix content indicated a certain degree of structural unfolding, suggesting that GLICL is a weak modifier of BSA’s secondary structure [41]. However, in the presented study, the α-helix content of gHSA_FRC_ increased from 20.00 ± 0.42% to 29.90 ± 0.14% upon interaction with LOS (Table 6). This result may indicate that LOS stabilizes the secondary structure of the protein glycated by fructose. This stabilization could result from forming new hydrogen bonds or other interactions between the ligand and albumin, leading to more significant structural organization and increased α-helix content. Based on the DSSP (Dictionary of Secondary Structure of Proteins) method, Moeinpour et al. (2016) observed that LOS subtly impacts hydrogen bonds and, consequently, the secondary structure of HSA [42]. It should be emphasized that studies using CD spectroscopy to investigate the secondary structure of proteins after glycation are inconclusive. Some researchers noted glucose-concentration-dependent changes in CD spectra, while others observed partial denaturation and structural disintegration [43]. The results presented in this study indicate that various sugars, as glycation inducers, can affect the secondary structure of human serum albumin differently.

## 3. Materials and Methods

### 3.1. Chemicals and Reagents

ImmunO Human serum albumin, fraction V, was purchased from MP Biomedicals LLC (Illkirch, France) and used without further purification; glipizide (GLP), losartan (LOS), sodium azide (NaN_3_), and dimethyl sulfoxide (DMSO) were provided by Sigma-Aldrich Chemical Co. (Darmstadt, Germany); D(-)-fructose (FRC) and D(+)-glucose (GLC) were obtained from POCH S.A. (Gliwice, Poland); di-Potassium hydrogen phosphate pure p.a. (K_2_HPO_4_) and sodium dihydrogen phosphate dehydrate (NaH_2_PO_4_ × 2H_2_O) were from Eurochem BGD Sp. z o.o. (Tarnów, Poland). All chemicals and reagents were of the highest analytical grade.

### 3.2. Methods

#### 3.2.1. Sample Preparation

HSA Glycation: Before the in vitro glycation of human serum albumin (HSA), all glassware and spatulas were sterilized to prevent bacterial contamination. Glycated albumins (gHSA_GLC_ and gHSA_FRC_) were prepared by exposing HSA solutions (5 × 10^−6^ mol∙L^−1^) to D(+)-glucose (GLC) (0.05 mol∙L^−1^) and D(-)-fructose (FRC) (0.05 mol∙L^−1^) in a phosphate buffer (0.05 mol∙L^−1^) preserved with sodium azide (0.015 mol∙L^−1^) for 21 days incubation at a temperature of t = 37 °C. A control sample (HSA) was prepared in the same manner, but without the addition of reducing sugars. After the incubation period, the non-glycated HSA and glycated albumins were extensively dialyzed against phosphate buffer (pH = 7.4 ± 0.1) for 24 h to remove the excess unbound GLC and FRC, then passed through a sterile Millex-GP syringe filter with 0.2 μm pores. The pH of the buffer solution, HSA, gHSA_GLC_, and gHSA_FRC_ was confirmed using a pH meter (FEP20 Mettler Toledo, Columbus, Ohio, USA). The absorbance ratio of HSA, gHSA_GLC_, and gHSA_FRC_ at λ = 255 nm and λ = 280 nm was less than 0.5, indicating the purity of the prepared albumin solutions.

Investigating the Interaction of Losartan (LOS) and Glipizide (GLP) with Non-glycated and Glycated Human Serum Albumins in Binary and Ternary Complexes: A quantitative analysis of the competition between LOS and GLP for the binding site in HSA, gHSA_GLC_, and gHSA_FRC_ was conducted using fluorimetric titration. To obtain the binary complexes of LOS and GLP with non-glycated and glycated albumins (LOS–HSA, LOS–gHSA_GLC_, LOS–gHSA_FRC_, and GLP–HSA, GLP–gHSA_GLC_, GLP–gHSA_FRC_), the albumin solutions (5 × 10^−6^ mol∙L^−1^) were titrated directly in the cuvette by adding increasing aliquots of LOS (5 × 10^−3^ mol∙L^−1^) and GLP (2.5 × 10^−3^ mol∙L^−1^) using a Hamilton syringe. The concentration range for LOS was from 5 × 10^−6^ mol∙L^−1^ to 5 × 10^−5^ mol∙L^−1^, and for GLP, it was from 2.5 × 10^−6^ mol∙L^−1^ to 2.5 × 10^−5^ mol∙L^−1^. To obtain the ternary complexes LOS–HSA–GLP_const_, LOS–gHSA_GLC_–GLP_const_, LOS–gHSA_FRC_–GLP_const_, GLP–HSA–LOS_const_, GLP–gHSA_GLC_–LOS_const_, and GLP–gHSA_FRC_–LOS_const_, the albumin solution (5 × 10^−6^ mol∙L^−1^) in the presence of GLP (5 × 10^−6^ mol·L^−1^) or LOS (5 × 10^−6^ mol·L^−1^) (molar ratio drug:albumin 1:1) was titrated in the cuvette by adding ten aliquots of LOS (5 × 10^−3^ mol∙L^−1^) or GLP (2.5 × 10^−3^ mol∙L^−1^), respectively. This procedure was conducted directly before fluorescence measurements. The ligand (GLP_const_ or LOS_const_) was preincubated with HSA, gHSA_GLC_, and gHSA_FRC_ for 30 min before adding the second corresponding ligand (LOS or GLP) to allow the ligand–albumin complexes to reach equilibrium. The LOS and GLP stock solutions were meticulously prepared by dissolving the appropriate amounts in distilled water and DMSO (ensuring DMSO did not exceed 1% *v*/*v* in the final concentration), respectively. 

Investigating Ligand Interactions—Spectrophotometric Analysis of LOS and GLP: The solutions of LOS and GLP at a concentration of 2.5 × 10^−5^ mol∙L^−1^, as well as the drug mixture (GLP + LOS; GLP:LOS molar ratio 1:1), were prepared in phosphate buffer (pH = 7.4 ± 0.1, 0.05 mol∙L^−1^), by diluting the stock solutions of the drugs (2.5 × 10^−3^ mol∙L^−1^). 

Spectropolarimetric Analysis of Glycation and LOS Influence on Macromolecule Secondary Structure: In this study, the samples (HSA, gHSA_GLC_, and gHSA_FRC_ at a concentration of 5 × 10^−6^ mol·L^−1^ each, as well as the LOS–HSA, LOS–gHSA_GLC_, LOS–gHSA_FRC_ complexes, where the molar ratio of LOS to albumin was 10:1) were prepared according to the procedure described above. 

#### 3.2.2. Fluorescence, UV-Vis, and Dichroism (CD) Spectra Measurements

The samples’ fluorescence spectra were measured at t = 37 °C using a JASCO FP-6500 spectrofluorimeter (Hachioji, Tokyo, Japan) equipped with a Peltier thermostat and standard quartz cells. The accuracy of the wavelength was ±1.5 nm. Excitation at λ_ex_ = 335 nm (λ_em_ = 350–500 nm) and λ_ex_ = 485 nm (λ_em_ = 500–580 nm) was used to measure the fluorescence of advanced glycation end products (AGEs) in non-glycated (HSA) and glycated human serum albumin (gHSA_GLC_, gHSA_FRC_). 

To excite the albumin fluorophores (Tyr and Trp-214 residues) in the binary (LOS–HSA, LOS–gHSA_GLC_, LOS–gHSA_FRC_, GLP–HSA, GLP–gHSA_GLC_, GLP–gHSA_FRC_) and ternary systems (LOS–HSA–GLP_const_, LOS–gHSA_GLC_–GLP_const_, LOS–gHSA_FRC_–GLP_const_, GLP–HSA–LOS_const_, GLP–gHSA_GLC_–LOS_const_, and GLP–gHSA_FRC_–LOS_const_), excitation wavelengths of λ_ex_ = 275 nm (excites Tyr and Trp-214 residues, λ_em_ = 285–400 nm) and λ_ex_ = 295 nm (excites Trp-214 residue, λ_em_ = 305–400 nm) were used. Finally, using JASCO software (Spectra Analysis, version 1.53.07), the light scattering spectrum of the buffer was subtracted from all fluorescence spectra. 

Due to the inner filter effect resulting from the presence of the drugs, the recorded fluorescence of HSA, gHSA_GLC_, and gHSA_FRC_ in the binary and ternary systems was corrected using Equation (1) [44]. This equation is applicable provided that the increase in absorbance within the system does not exceed approximately 0.3.

(1) $$F_{cor}=F_{obs}\cdot e^{\left(\frac{{Abs}_{ex}+{Abs}_{em}}{2}\right)}$$

where $F_{cor}$ and $F_{obs}$ are the corrected and observed fluorescence intensities, respectively; and ${Abs}_{ex}$ and ${Abs}_{em}$ are the absorbance at the excitation and emission wavelengths, respectively. 

The samples’ absorbance measurements were recorded using a model V-760 JASCO spectrophotometer (Easton, MD, USA) equipped with a thermostat bath, using quartz cuvettes of dimensions 1.0 cm × 1.0 cm × 4.0 cm. The apparatus has a wavelength correction error of ±0.3 nm and a photometric correction error of ±0.002 Abs at 0.5 Abs.

The absorption spectra of GLP and LOS at a concentration of 2.5 × 10^−5^ mol∙L^−1^, as well as the GLP + LOS system at GLP:LOS 1:1 (*v*/*v*) molar ratio, were determined in the wavelength range of λ = 235–300 nm at t = 37 °C. 

Far-UV CD spectra of HSA, gHSA_GLC_, gHSA_FRC_, and LOS–HSA, LOS–gHSA_GLC_, and LOS–gHSA_FRC_ complexes were recorded using a JASCO model J-1500 CD spectropolarimeter (Hachioji, Tokyo, Japan) equipped with a thermostatic Peltier cell holder (accuracy t = ±0.05 °C). All spectra were measured in a 0.1 cm path length quartz cuvette and scanned from λ = 200 to 250 nm at wavelength intervals of 0.2 nm, with a bandwidth set at 2.0 nm and a D.I.T. of 4 s. CD intensity was expressed as mean residue ellipticity at wavelength λ (${\left[\theta \right]}_{mrw}$) (deg·cm^2^·dmol^−1^) according to Equation (2) [38]:

(2) $${\left[\theta \right]}_{mrw}=\frac{MRW\cdot \theta_{\lambda }}{10\cdot d\cdot c}$$

where MRW is the mean residue weight (${MRW}_{HSA}=113.7$ Da); $\theta_{\lambda }$ is the observed ellipticity at wavelength λ (deg); d is the optical path length (cm); c is the protein concentration (g·cm^−3^). 

The content of the samples’ secondary structure elements was calculated using the Secondary Structure Estimation program (version 2.13.00) with Yang’s reference model.

The fluorescence, absorption, and Far-UV CD spectra presented in this study were corrected by smoothing using the Savitzky and Golay method with a convolution width of 15, using the Spectra Analysis program (version 1.53.07, JASCO, Easton, MD, USA). 

Based on the calculated fluorescence emission intensities, the quenching curves ($\frac{F}{F_{0}}$ vs. drug:albumin molar ratio, where F, $F_{0}$ are the fluorescence intensities at the maximum wavelength in the presence and absence of the quencher, respectively) of non-glycated and glycated human serum albumin in the presence of losartan (LOS) or glipizide (GLP) (binary system: LOS–HSA, LOS–gHSA_GLC_, LOS–gHSA_FRC_ and GLP–HSA, GLP–gHSA_GLC_, GLP–gHSA_FRC_) or in the presence of both drugs (ternary system: LOS–HSA–GLP_const_, LOS–gHSA_GLC_–GLP_const_, LOS–gHSA_FRC_–GLP_const_ and GLP–HSA–LOS_const_, GLP–gHSA_GLC_–LOS_const_, GLP–gHSA_FRC_–LOS_const_) were plotted.

The fluorescence quenching mechanism (static and/or dynamic) of HSA, gHSA_GLC_, and gHSA_FRC_ in the absence or presence of LOS and GLP in the binary and ternary systems was analyzed based on the Stern–Volmer equation (Equation (3)) [29]:

(3) $$\frac{F_{0}}{F}=1+k_{q}\tau_{0}\cdot \left[L\right]=1+K_{SV}\cdot [L]$$

where $k_{q}$ is the bimolecular quenching rate constant (L∙mol^−1^∙s^−1^), $k_{q}=\frac{K_{SV}}{\tau_{0}}$; $\tau_{0}$ is the average fluorescence lifetime of albumin without a quencher, $\tau_{0}$ = 6.0 × 10^−9^ s [28]; $K_{SV}$ is the Stern–Volmer constant (L∙mol^−1^); $\left[L\right]$ is the ligand concentration (mol∙L^−1^); $\left[L\right]=\left[L_{b}\right]+\left[L_{f}\right]$, where $\left[L_{b}\right]$ and $\left[L_{f}\right]$ are the bound and unbound (free) drug concentrations, respectively.

The association constant ${(K}_{a})$ and the number of binding sites classes (n) in the ligand–albumin or ligand–albumin–ligand_const_ complexes were determined using the Scatchard (Equation (4)) [45] and Klotz (Equation (5)) equations [46]:

(4) $$\frac{r}{{[L}_{f}]}=n\cdot K_{a}-K_{a}\cdot r$$

(5) $$\frac{1}{r}=\frac{1}{n}+\frac{1}{n\cdot K_{a}\cdot {[L}_{f}]}$$

where $r=\frac{{[L}_{b}]}{\left[P\right]}$ is the number of ligand moles bound to one mole of protein; $\left[P\right]$ is the total protein concentration (mol∙L^−1^); $\left[L_{b}\right]=\frac{∆F}{{∆F}_{max}}\cdot [P]$, ${∆F}_{max}$ is maximal fluorescence change with complete saturation (evaluated from the linear part of the $\frac{1}{∆F}vs.\frac{1}{\left[L\right]}$), and ∆F is the difference between $F_{0}$ and F.

For two classes of binding sites in albumin structure (in this study, GLP–HSA and LOS–gHSA_FRC_–GLP_const_ complexes), the binding isotherms were drawn using non-linear regression analysis according to Equation (6), and the association constants ${(K}_{a1},\;K_{a2})$ and the number of binding sites ${(n}_{1},\;n_{2})$ were calculated [47]:

(6) $$r=\frac{n_{1}\cdot K_{a1}\cdot \left[L_{f}\right]}{1+K_{a1}\cdot \left[L_{f}\right]}+\frac{n_{2}\cdot K_{a2}\cdot \left[L_{f}\right]}{1+K_{a2}\cdot \left[L_{f}\right]}$$

Hill’s coefficient $n_{H}$ was determined on the basis of the Hill method (Equation (6)) [35]:

(7) $$log\left(\frac{r}{1-r}\right)=n_{H}\cdot log{[L}_{f}]+logK_{a}$$

### 3.3. Statistics

The experiments were conducted in triplicate, and the results are presented as the mean ± relative standard deviation (mean ± RSD). Linear regression analysis (R^2^) was performed by fitting the experimental data to the appropriate equation using OriginPro version 8.5 SR1 (Northampton, MA, USA). 

## 4. Conclusions

The main goal of this project was to analyze the interactions of losartan (LOS) and glipizide (GLP) with non-glycated (HSA) and glycated human serum albumin (gHSA_GLC_ and gHSA_FRC_) and to investigate their mutual competition in the binding sites of the macromolecule using multiple spectroscopic techniques. 

The analysis of the emission fluorescence spectra of glycation products (AGEs) indicated that fructose (FRC) is a more effective glycation agent for HSA than glucose (GLC). This conclusion is supported by the observation that glycation in the presence of FRC leads to a faster and more efficient Amadori rearrangement. Glycation induces conformational changes in the albumin structure, particularly altering the microenvironment around crucial amino acid residues (Trp-214 and Tyr), thus influencing their accessibility to quenchers. The results showed that LOS and GLP had a higher affinity for non-glycated HSA than glycated gHSA_GLC_ and gHSA_FRC_, forming a more stable complex. Additionally, LOS and GLP were found to interact nonspecifically with the hydrophobic fragments of the surface of albumins in binary (ligand–albumin) and ternary systems (ligand–albumin–ligand_const_), and to specifically saturate the binding sites within the protein molecule. An additional drug (GLP in the LOS–albumin complex or LOS in the GLP–albumin complex) complicated the interaction, likely leading to competitive binding or displacement of the initially bound drug in both non- and glycated albumins. Analysis of the UV spectra revealed that GLP and LOS interact with each other. This finding emphasizes the importance of closely monitoring patients co-administered these drugs, focusing on blood glucose levels, blood pressure, and renal function, to ensure safe and effective therapy. 

In conclusion, the significant impact of glycation on the drug-binding capacity of albumin, as revealed by this study, has profound implications for patient care. Particularly for diabetic patients, the altered binding properties of glycated albumin could potentially compromise the efficacy and safety of drug therapy. Therefore, the need for careful monitoring, and potentially adjusted dosing regimens, is urgent, to ensure optimal therapeutic outcomes. Further clinical studies are imperative, to develop comprehensive guidelines for the concurrent use of LOS and GLP in polypharmacotherapy.

## Figures and Tables

**Table 1:** Stern–Volmer constants $K_{SV}$ (L∙mol^−1^) calculated for LOS–HSA, LOS–gHSA_GLC_, LOS–gHSA_FRC_, and GLP–HSA, GLP–gHSA_GLC_, GLP–gHSA_FRC_, LOS–HSA–GLP_const_, LOS–gHSA_GLC_–GLP_const_, LOS–gHSA_FRC_–GLP_const_, and GLP–HSA–LOS_const_, GLP–gHSA_GLC_–LOS_const_, GLP–gHSA_FRC_–LOS_const_; λ_ex_ = 275 nm and λ_ex_ = 295 nm.

λ_ex_ = 275 nm	$K_{SV}$ ± RSD * × 10^4^ (L∙mol^−1^)	λ_ex_ = 275 nm	$K_{SV}$ ± RSD * × 10^4^ (L∙mol^−1^)
LOS-HSA	1.90 ± 0.02	GLP-HSA	5.22 ± 0.10
LOS-gHSA_GLC_	1.40 ± 0.02	GLP-gHSA_GLC_	3.78 ± 0.08
LOS–gHSA_FRC_	0.82 ± 0.02	GLP–gHSA_FRC_	2.47 ± 0.09
LOS–HSA–GLP_const_	1.18 ± 0.03	GLP–HSA–LOS_const_	3.50 ± 0.07
LOS–gHSA_GLC_–GLP_const_	1.04 ± 0.04	GLP–gHSA_GLC_–LOS_const_	3.61 ± 0.13
LOS–gHSA_FRC_–GLP_const_	0.77 ± 0.06	GLP–gHSA_FRC_–LOS_const_	2.36 ± 0.18
λ_ex_ = 295 nm	$K_{SV}$ ± RSD * × 10^4^ (L∙mol^−1^)	λ_ex_ = 295 nm	$K_{SV}$ ± RSD * × 10^4^ (L∙mol^−1^)
LOS–HSA	1.61 ± 0.04	GLP–HSA	3.96 ± 0.17
LOS–gHSA_GLC_	0.97 ± 0.02	GLP–gHSA_GLC_	2.24 ± 0.10
LOS–gHSA_FRC_	0.39 ± 0.01	GLP–gHSA_FRC_	1.03 ± 0.07
LOS–HSA–GLP_const_	0.76 ± 0.04	GLP–HSA–LOS_const_	1.94 ± 0.07
LOS–gHSA_GLC_–GLP_const_	0.58 ± 0.04	GLP–gHSA_GLC_–LOS_const_	2.15 ± 0.16
LOS–gHSA_FRC_–GLP_const_	0.28 ± 0.05	GLP–gHSA_FRC_–LOS_const_	0.99 ± 0.14

* Relative standard deviation.

**Table 2:** Association constants $K_{a}$ (L∙mol^−1^), mean number of LOS molecules bound with one molecule of HSA, gHSA_GLC_, and gHSA_FRC_ (n), the Hill’s coefficient $\left(n_{H}\right)$ in the binary (LOS–HSA, LOS–gHSA_GLC_, LOS–gHSA_FRC_) and ternary system (LOS–HSA–GLP_const_, LOS–gHSA_GLC_–GLP_const_, LOS–gHSA_FRC_–GLP_const_); λ_ex_ = 275 nm and λ_ex_ = 295 nm.

	Scatchard Method		Klotz Method		Hill Method
λ_ex_ = 275 nm	$K_{a}$ ± RSD * × 10^4^ (L∙mol^−1^)	n ± RSD *	$K_{a}$ ± RSD * × 10^4^ (L∙mol^−1^)	n ± RSD *	$n_{H}$ ± RSD *
LOS–HSA	3.66 ± 0.09	0.92 ± 0.01	3.69 ± 0.03	0.93 ± 0.01	0.94 ± 0.02
LOS–gHSA_GLC_	2.45 ± 0.13	1.12 ± 0.03	3.25 ± 0.16	0.93 ± 0.08	1.04 ± 0.03
LOS–gHSA_FRC_	1.05 ± 0.18	1.08 ± 0.08	1.17 ± 0.11	0.97 ± 0.07	1.02 ± 0.04
LOS–HSA–GLP_const_	1.19 ± 0.01	1.11 ± 0.01	1.37 ± 0.05	0.98 ± 0.08	1.02 ± 0.03
LOS–gHSA_GLC_–GLP_const_	1.10 ± 0.05	1.01 ± 0.04	1.14 ± 0.03	1.01 ± 0.04	1.00 ± 0.01
LOS–gHSA_FRC_–GLP_const_	0.87 ± 0.03	1.71 ± 0.10	0.95 ± 0.03	1.00 ± 0.03	1.00 ± 0.01
λ_ex_ = 295 nm	$K_{a}$ ± RSD * × 10^4^ (L∙mol^−1^)	n ± RSD *	$K_{a}$ ± RSD * × 10^4^ (L∙mol^−1^)	n ± RSD *	$n_{H}$ ± RSD *
LOS–HSA	3.38 ± 0.10	0.95 ± 0.02	3.43 ± 0.07	0.94 ± 0.03	0.95 ± 0.01
LOS–gHSA_GLC_	2.51 ± 0.16	1.09 ± 0.04	3.25 ± 0.14	0.89 ± 0.10	1.06 ± 0.03
LOS–gHSA_FRC_	0.98 ± 0.08	1.03 ± 0.08	1.09 ± 0.10	0.97 ± 0.10	1.02 ± 0.01
LOS–HSA–GLP_const_	1.16 ± 0.10	0.92 ± 0.10	1.18 ± 0.09	0.92 ± 0.03	0.99 ± 0.01
LOS–gHSA_GLC_–GLP_const_	1.10 ± 0.05	1.01 ± 0.04	1.13 ± 0.06	0.98 ± 0.07	1.02 ± 0.04
LOS–gHSA_FRC_–GLP_const_	^(a)^ 0.55 ± 0.03	^(a)^ 0.85 ± 0.01	^(b)^ -	^(b)^ -	1.20 ± 0.10

* Relative standard deviation; ^(a)^ determined by non-linear regression using binding isotherms; ^(b)^ impossible to determine.

**Table 3:** Association constants $K_{a}$ (L∙mol^−1^), mean number of GLP molecules bound with one molecule of HSA, gHSA_GLC_, and gHSA_FRC_ (n), the Hill’s coefficient $\left(n_{H}\right)$ in the binary (GLP–HSA, GLP–gHSA_GLC_, GLP–gHSA_FRC_) and ternary system (GLP–HSA–LOS_const_, GLP–gHSA_GLC_–LOS_const_, GLP–gHSA_FRC_–LOS_const_); λ_ex_ = 275 nm and λ_ex_ = 295 nm.

	Scatchard Method		Klotz Method		Hill Method
λ_ex_ = 275 nm	$K_{a}$ ± RSD * × 10^4^ (L∙mol^−1^)	n ± RSD *	$K_{a}$ ± RSD * × 10^4^ (L∙mol^−1^)	n ± RSD *	$n_{H}$ ± RSD *
GLP–HSA	^(a)^ 53.72 ± 3.41	^(a)^ 0.45 ± 0.06	23.21 ± 0.06	0.87 ± 0.02	0.92 ± 0.03
	^(a)^ 3.67 ± 0.23	^(a)^ 0.92 ± 0.04			
GLP–gHSA_GLC_	6.65 ± 0.18	0.93 ± 0.01	7.45 ± 0.17	0.86 ± 0.03	0.96 ± 0.03
GLP–gHSA_FRC_	3.25 ± 0.17	1.03 ± 0.01	3.62 ± 0.12	0.93 ± 0.05	1.00 ± 0.01
GLP–HSA–LOS_const_	3.99 ± 0.08	0.91 ± 0.02	4.12 ± 0.06	0.89 ± 0.02	0.97 ± 0.03
GLP–gHSA_GLC_–LOS_const_	6.58 ± 0.09	0.94 ± 0.04	7.20 ± 0.09	0.92 ± 0.04	0.96 ± 0.04
GLP–gHSA_FRC_–LOS_const_	2.43 ± 0.09	0.92 ± 0.07	2.32 ± 0.09	0.96 ± 0.08	0.99 ± 0.02
λ_ex_ = 295 nm	$K_{a}$ ± RSD * × 10^4^ (L∙mol^−1^)	n ± RSD *	$K_{a}$ ± RSD * × 10^4^ (L∙mol^−1^)	n ± RSD *	$n_{H}$ ± RSD *
GLP–HSA	^(a)^ 95.01 ± 21.03	^(a)^ 0.28 ± 0.03	34.21 ± 0.18	0.83 ± 0.03	0.84 ± 0.03
	^(a)^ 7.32 ± 1.02	^(a)^ 0.73 ± 0.05			
GLP–gHSA_GLC_	8.04 ± 0.46	0.91 ± 0.01	9.03 ± 0.09	0.87 ± 0.02	0.96 ± 0.04
GLP–gHSA_FRC_	2.39 ± 0.22	0.93 ± 0.08	3.03 ± 0.10	0.90 ± 0.07	1.01 ± 0.01
GLP–HSA–LOS_const_	5.04 ± 0.19	0.95 ± 0.06	5.72 ± 0.15	0.91 ± 0.03	0.99 ± 0.03
GLP–gHSA_GLC_–LOS_const_	6.68 ± 0.16	0.91 ± 0.05	7.32 ± 0.09	0.94 ± 0.04	0.94 ± 0.01
GLP–gHSA_FRC_–LOS_const_	2.95 ± 0.14	0.92 ± 0.04	2.72 ± 0.13	0.96 ± 0.05	0.97 ± 0.03

* Relative standard deviation; ^(a)^ determined by non-linear regression using binding isotherms.

**Table 4:** The values of maximum absorbance of GLP, LOS, and drug mixture at the selected wavelengths λ = 235 nm, λ = 253 nm, and λ = 282 nm.

λ (nm)	Absorbance ± RSD * (a.u.)			Mathematic Sum of GLP and LOS Absorbance ± RSD *
	GLP	LOS	GLP + LOS	
235	0.9319 ± 0.0009	1.9768 ± 0.0018	2.5215 ± 0.0038	2.9087 ± 0.0027
253	0.1545 ± 0.0004	1.2960 ± 0.0041	1.4173 ± 0.0054	1.4505 ± 0.0045
282	0.2549 ± 0.0006	0.2456 ± 0.0016	0.4509 ± 0.0013	0.5005 ± 0.0024

* Relative standard deviation.

**Table 5:** The values of non-glycated and glycated albumin mean residue ellipticity ${\left[\theta \right]}_{mrw}$ in the sample without (HSA, gHSA_GLC_, gHSA_FRC_) and in the presence of losartan (LOS-HSA, LOS- gHSA_GLC_, LOS-gHSA_FRC_).

Sample **	λ_min_ (nm)	${\left[\theta \right]}_{mrw}$ ± RSD * (deg·cm^2^·dmol^−1^)	HT ± RSD * (V)
HSA	209.2	−13,855.23 ± 58.24	281.61 ± 0.04
	221.2	−13,087.41 ± 87.13	242.06 ± 0.09
gHSA_GLC_	208.8	−12,324.99 ± 113.28	284.85 ± 0.06
	221.2	−11,529.40 ± 116.45	242.88 ± 0.10
gHSA_FRC_	208.6	−11,871.22 ± 328.16	286.79 ± 0.08
	221.2	−10,453.40 ± 342.24	243.58 ± 0.04
LOS–HSA	209.2	−13,869.50 ± 131.10	301.08 ± 0.10
	221.2	−13,035.94 ± 182.71	254.15 ± 0.07
LOS–gHSA_GLC_	208.8	−11,993.66 ± 95.42	298.99 ± 0.01
	221.2	−11,273.80 ± 103.28	251.65 ± 0.05
LOS–gHSA_FRC_	208.6	−11,513.06 ± 242.21	300.09 ± 0.08
	221.2	−10,230.25 ± 208.15	253.13 ± 0.09

* Relative standard deviation; ** for the remaining complexes (GLP–HSA, GLP–gHSA_GLC_, GLP–gHSA_FRC_, GLP–HSA–LOS_const,_ GLP–gHSA_GLC_–LOS_const_, GLP–gHSA_FRC_–LOS_const_, LOS–HSA–GLP_const_, LOS–gHSA_GLC_–GLP_const_, LOS–gHSA_FRC_–GLP_const_), obtaining CD spectra was impossible due to excessively weak signals, likely caused by too low a concentration of GLP. This resulted in the sample absorbance exceeding the detector’s capacity, leading to off-scale signals—HT (high-tension) readings outside the acceptable range.

**Table 6:** The percentage content (%) of non-glycated and glycated albumin secondary structure elements in the sample without (HSA, gHSA_GLC_, gHSA_FRC_) and in the presence of losartan (LOS–HSA, LOS–gHSA_GLC_, LOS–gHSA_FRC_) based on Yang’s reference model.

	% α-Helix ± RSD *	% β-Sheet ± RSD *	% Turn ± RSD *	% Random ± RSD *
HSA	36.65 ± 0.58	10.20 ± 0.26	22.65 ± 0.04	30.60 ± 0.14
gHSA_GLC_	35.70 ± 0.13	11.10 ± 0.14	22.40 ± 0.08	30.80 ± 0.14
gHSA_FRC_	20.00 ± 0.42	16.75 ± 0.05	21.65 ± 0.13	32.70 ± 0.17
LOS–HSA	35.75 ± 0.21	8.80 ± 0.16	23.95 ± 0.22	31.75 ± 0.15
LOS–gHSA_GLC_	35.85 ± 0.28	9.75 ± 0.22	23.45 ± 0.18	31.10 ± 0.18
LOS–gHSA_FRC_	29.90 ± 0.14	15.90 ± 0.45	21.90 ± 0.14	32.45 ± 0.13

* Relative standard deviation.

## Data Availability

The data underlying the findings of this study are accessible upon reasonable request to the author.

## Supplementary Materials

The following supporting information can be downloaded at https://www.mdpi.com/article/10.3390/ijms25179698/s1.
