# Supplementary material for: In Vitro Spectroscopic Investigation of Losartan and Glipizide Competitive Binding to Glycated Albumin: A Comparative Study
Source: Int J Mol Sci. 2024 Sep 7;25(17):9698. doi: 10.3390/ijms25179698 (PMC11395140; doi:10.3390/ijms25179698)
Supplement: Supplementary file 1 [file ijms-25-09698-s001.zip › ijms-3164737-supplementary.pdf]

Supplementary Materials

In Vitro Spectroscopic Investigation of Losartan and Glipizide Competitive Binding to Glycated Albumin: A Comparative Study

Agnieszka Szkudlarek

Department of Physical Pharmacy, Faculty of Pharmaceutical Sciences in Sosnowiec, Medical University of Silesia in Katowice, 40-055 Katowice, Poland; aszkudlarek@sum.edu.pl; Tel.: +48-32-364-1597

Table S1. The percentage (%) of fluorescence quenching of non-glycated (HSA) and glycated albumin ($gHSA_{GLC}$, $gHSA_{FRC}$) in the presence of losartan (LOS) and glipizide (GLP) with increasing concentration; λ_{ex} = 275 nm and λ_{ex} = 295 nm.

		λ_{ex} = 275 nm	λ_{ex} = 295 nm
**Ligand:Protein Molar Ratio**	**Molar Ratio**	**Percentage (%) of Fluorescence Quenching ± RSD ***	
LOS:HSA	0:1–10:1	49.20 ± 0.32	45.17 ± 0.77
LOS:$gHSA_{GLC}$		41.67 ± 0.21	33.36 ± 0.19
LOS:$gHSA_{FRC}$		29.62 ± 0.39	16.54 ± 0.24
GLP:HSA	0:1–5:1	57.86 ± 0.38	51.46 ± 0.82
GLP:$gHSA_{GLC}$		49.42 ± 0.45	37.41 ± 0.68
GLP:$gHSA_{FRC}$		38.93 ± 0.54	21.46 ± 0.86

* Relative standard deviation.

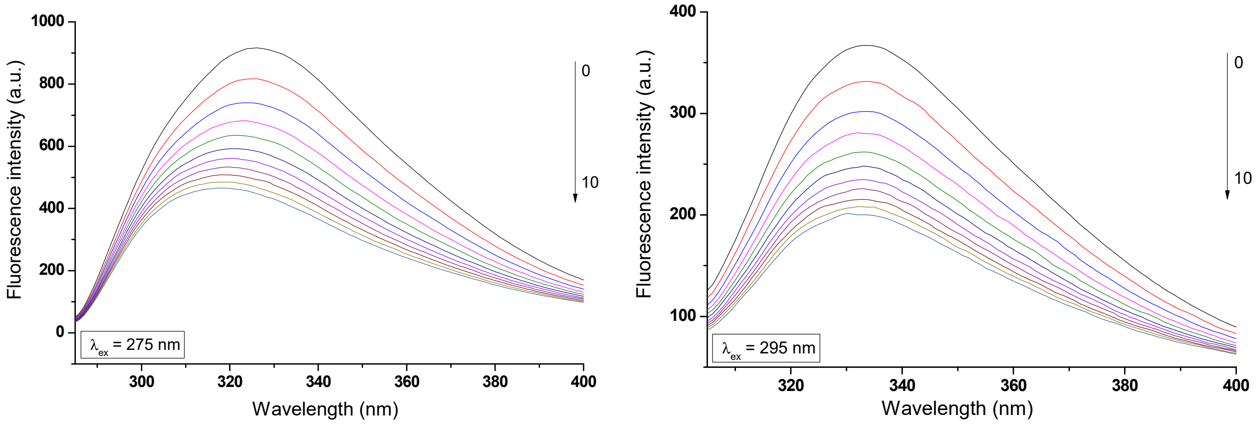

(**a**) (**b**)

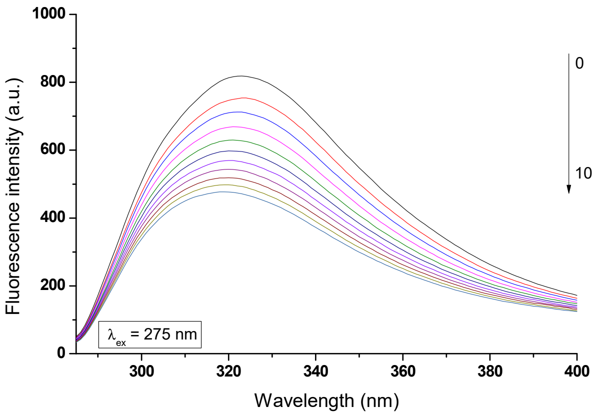

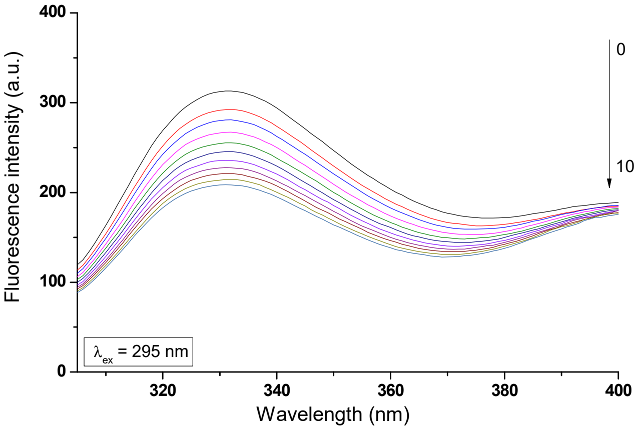

(**c**) (**d**)

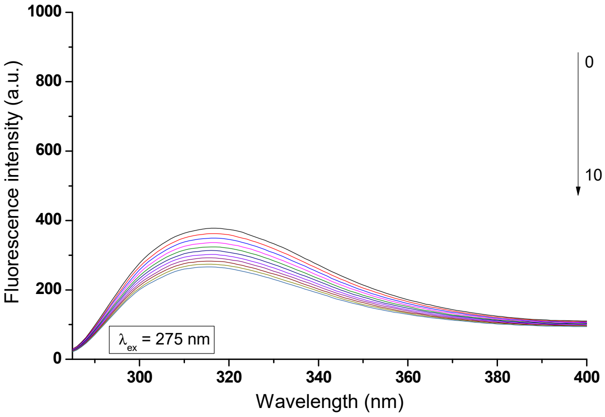

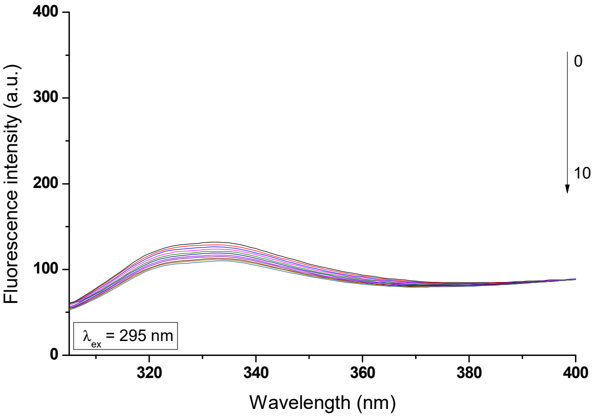

(**e**) (**f**)

Figure S1. Emission fluorescence spectra of (**a**,**b**) HSA, (**c**,**d**) $gHSA_{GLC}$ and (**e**,**f**) $gHSA_{FRC}$ at 5×10^{-6} mol·L^{-1} (0) concentration in the presence of increasing ligand concentrations (LOS at 5×10^{-6} mol·L^{-1} (1) to 5×10^{-5} mol·L^{-1} (10)); λ_{ex} = 275 nm and λ_{ex} = 295 nm; t = 37°C.

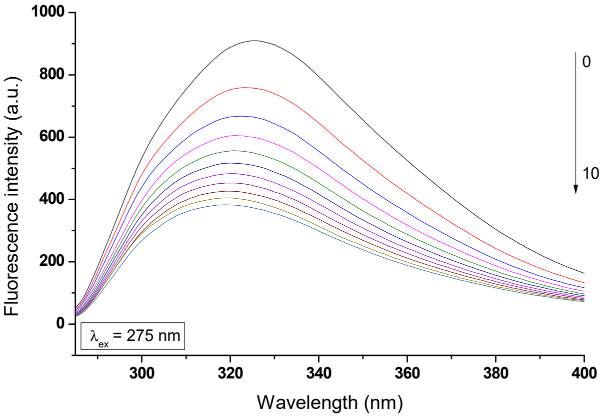

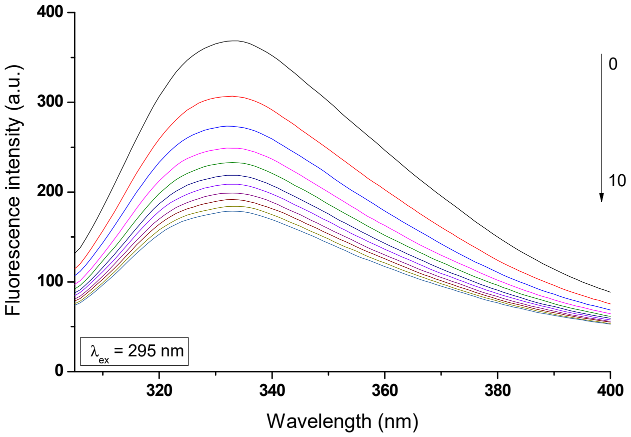

(**a**) (**b**)

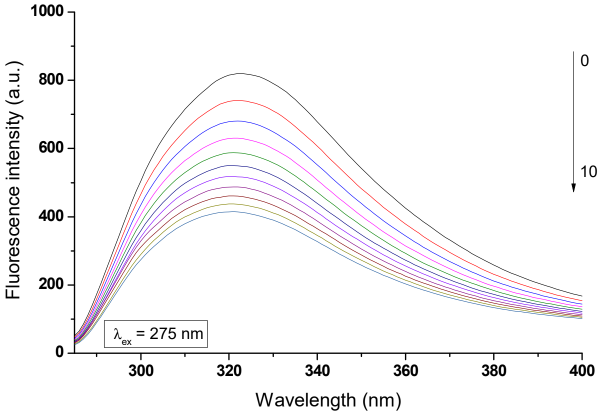

(**c**)

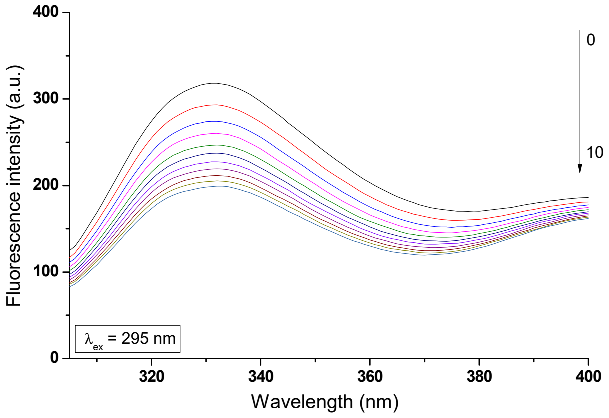

(**d**)

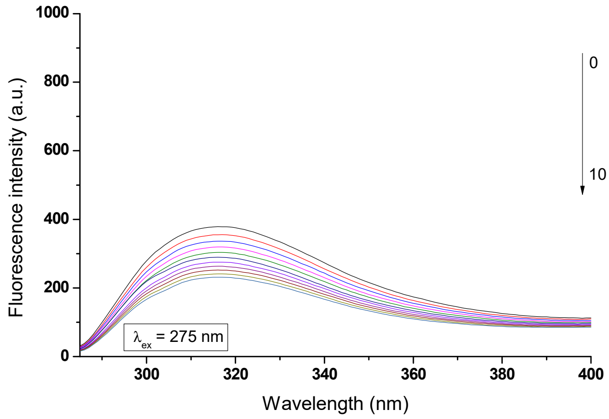

(**e**)

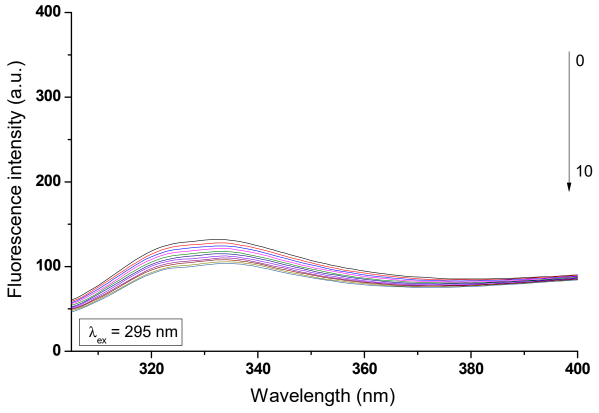

(**f**)

Figure S2. Emission fluorescence spectra of (**a**,**b**) HSA, (**c**,**d**) $gHSA_{GLC}$ and (**e**,**f**) $gHSA_{FRC}$ at 5×10^{-6} $mol \cdot L^{-1}$ (0) concentration in the presence of increasing ligand concentrations (GLP at 2.5×10^{-6} $mol \cdot L^{-1}$ (1) to 2.5×10^{-5} $mol \cdot L^{-1}$ (10)); $\lambda_{ex} = 275$ nm and $\lambda_{ex} = 295$ nm; t = 37°C.

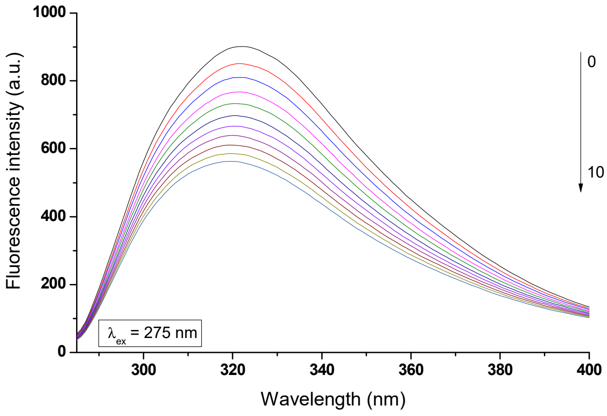

(**a**)

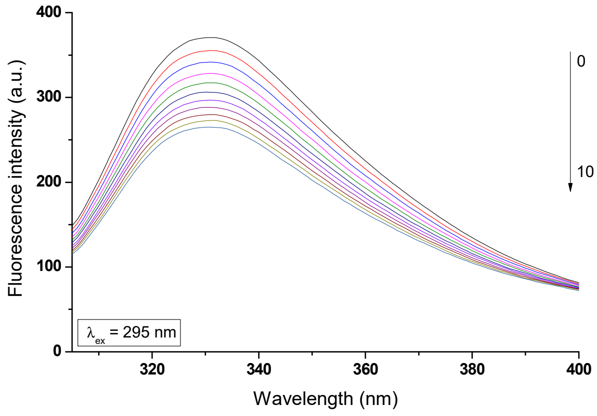

(**b**)

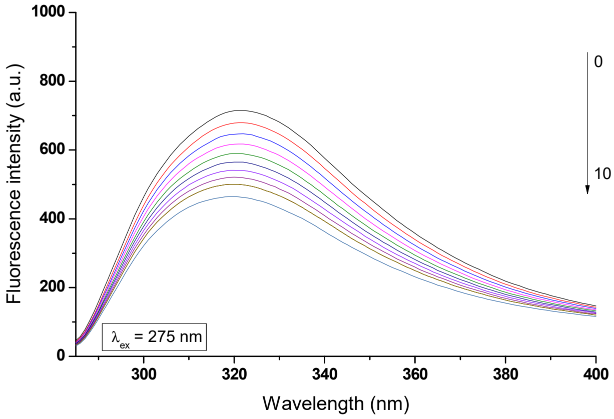

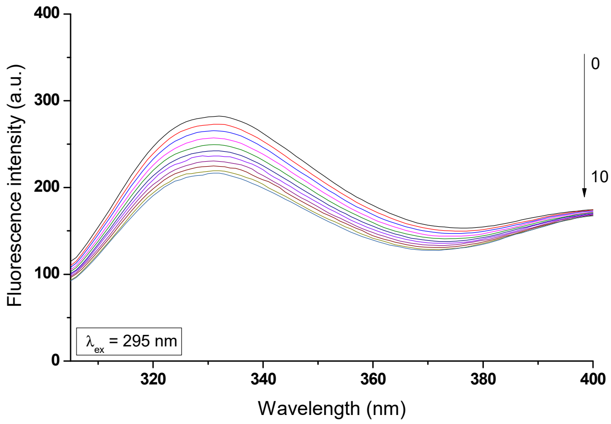

(**c**) (**d**)

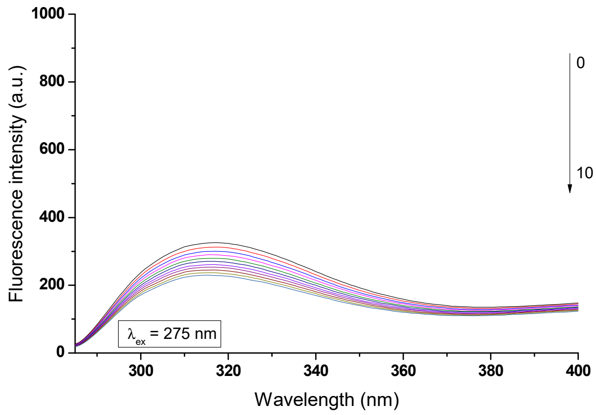

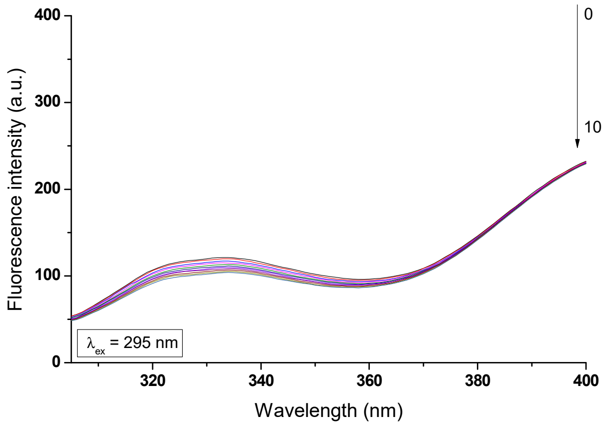

(**e**) (**f**)

Figure S3. Emission fluorescence spectra of (**a**, **b**) HSA-GLP$_{const}$, (**c**, **d**) gHSA$_{GLC}$-GLP$_{const}$ and (**e**, **f**) gHSA$_{FRC}$-GLP$_{const}$ at 5×10^{-6} mol·L^{-1} (0) concentration (molar ratio albumin:GLP 1:1) in the presence of increasing ligand concentrations (LOS at 5×10^{-6} mol·L^{-1} (1) to 5×10^{-5} mol·L^{-1} (10)); λ_{ex} = 275 nm and λ_{ex} = 295 nm; t = 37°C.

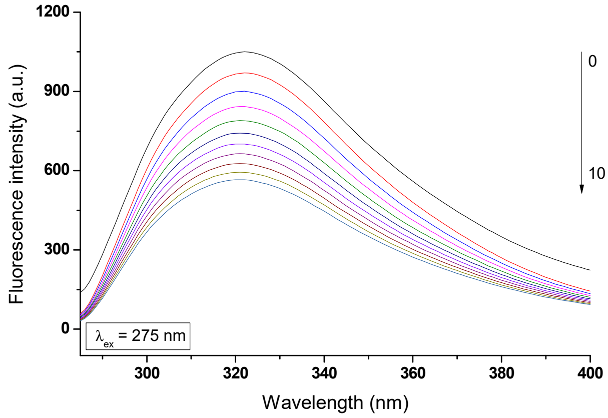

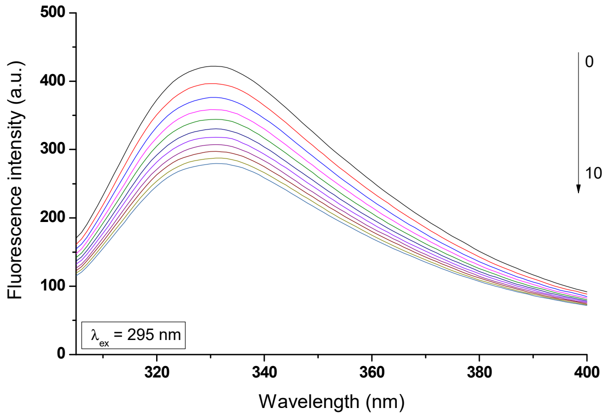

(**a**) (**b**)

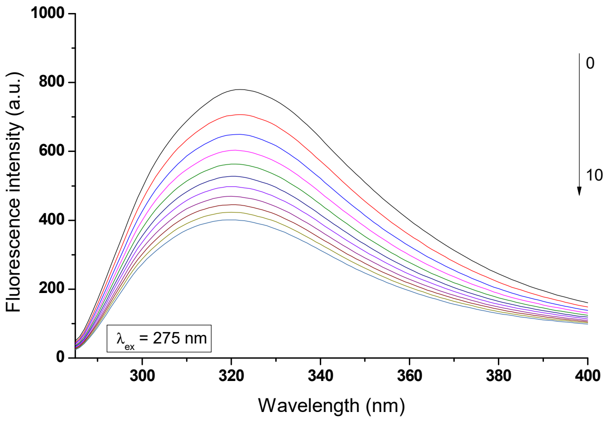

(**c**)

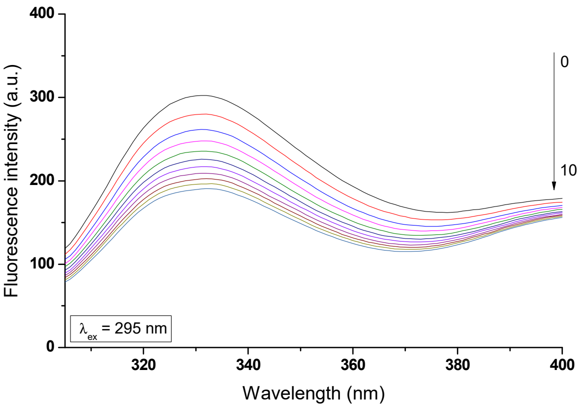

(**d**)

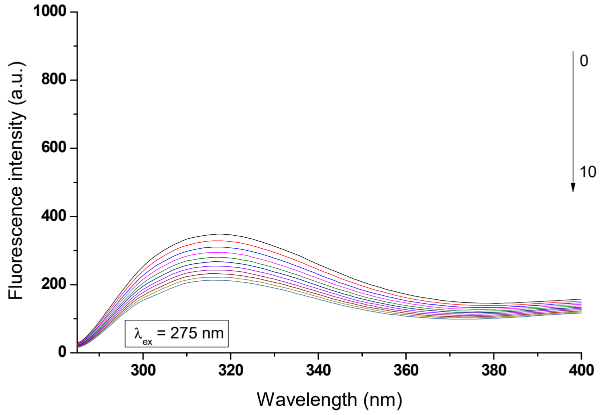

(**e**)

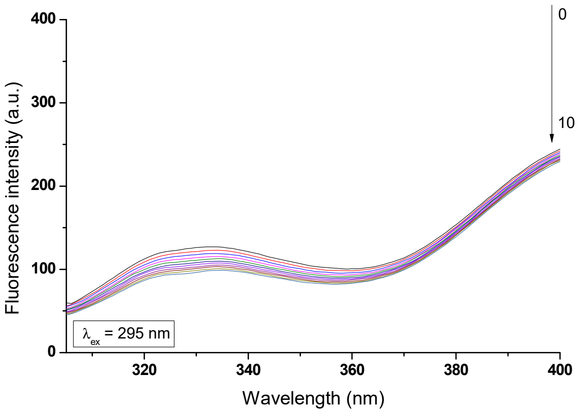

(**f**)

Figure S4. Emission fluorescence spectra of (**a**, **b**) HSA-LOS$_{const}$, (**c**, **d**) gHSA$_{GLC}$-LOS$_{const}$ and (**e**, **f**) gHSA$_{FRC}$-LOS$_{const}$ at 5×10^{-6} mol·L^{-1} (0) concentration (molar ratio albumin:LOS 1:1) in the presence of increasing ligand concentrations (GLP at 2.5×10^{-6} mol·L^{-1} (1) to 2.5×10^{-5} mol·L^{-1} (10)); λ_{ex} = 275 nm and λ_{ex} = 295 nm; t = 37°C.

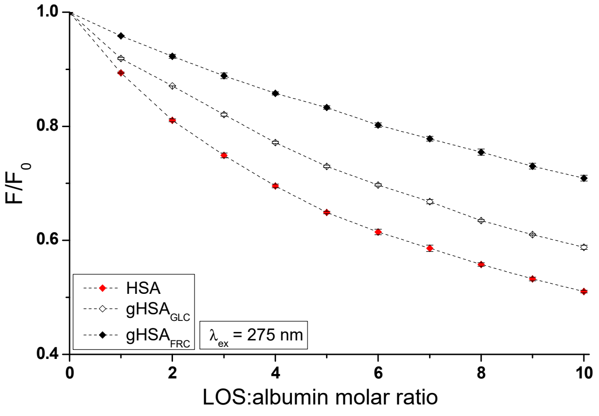

(**a**)

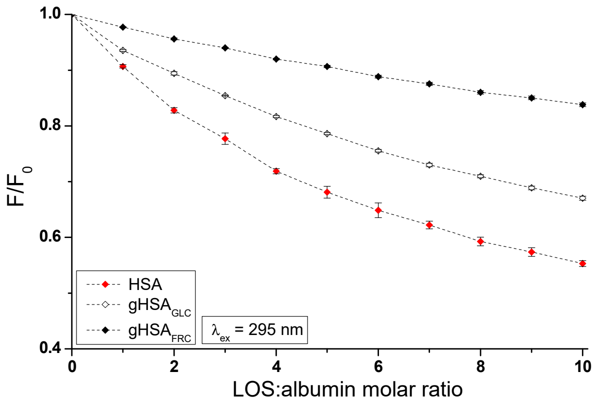

(**b**)

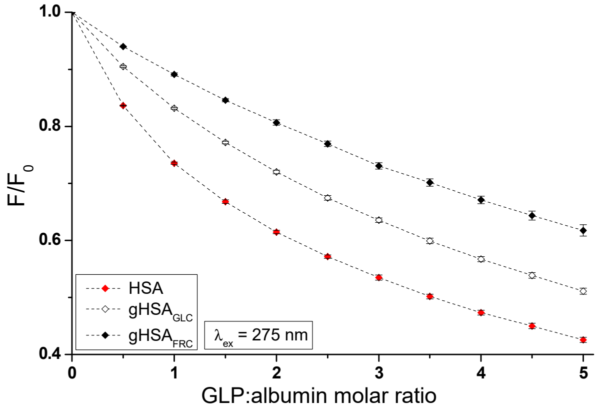

(c)

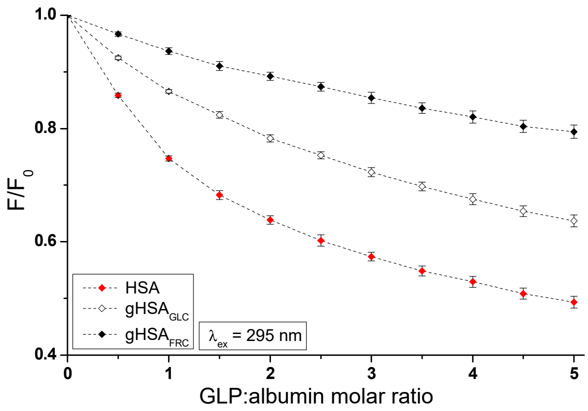

(d)

Figure S5. Quenching fluorescence of albumin non-glycated (HSA, ♦) and glycated ($gHSA_{GLC}$, ◊; $gHSA_{FRC}$, ♦) containing 5 × 10^{-6} mol·L^{-1} to 5 × 10^{-5} mol·L^{-1} concentrations of LOS (**a**,**b**) and 2.5 × 10^{-6} mol·L^{-1} to 2.5 × 10^{-5} mol·L^{-1} concentrations of GLP (**c**,**d**). Albumin concentration: 5 × 10^{-6} mol·L^{-1}; λ_{ex} = 275 nm (**a**, **c**) and λ_{ex} = 295 nm (**b**,**d**) .

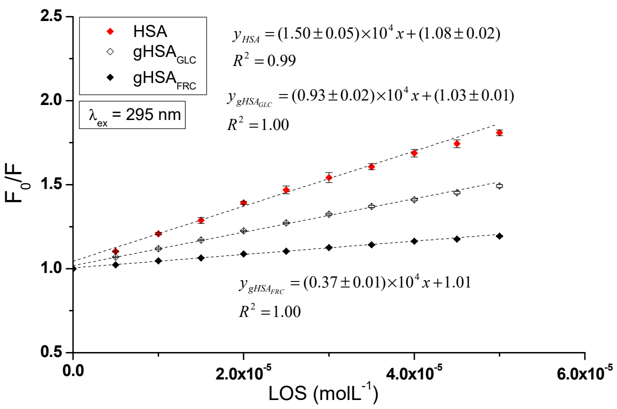

(**a**)

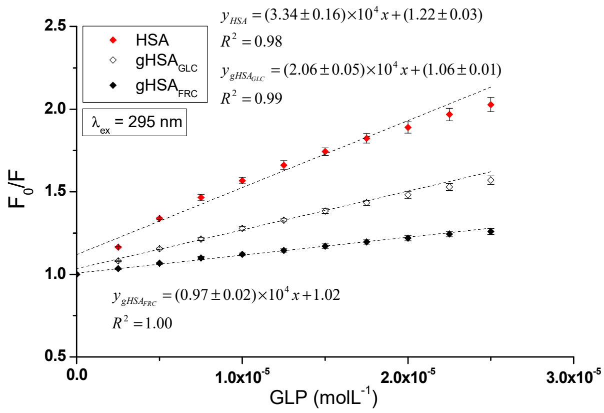

(**b**)

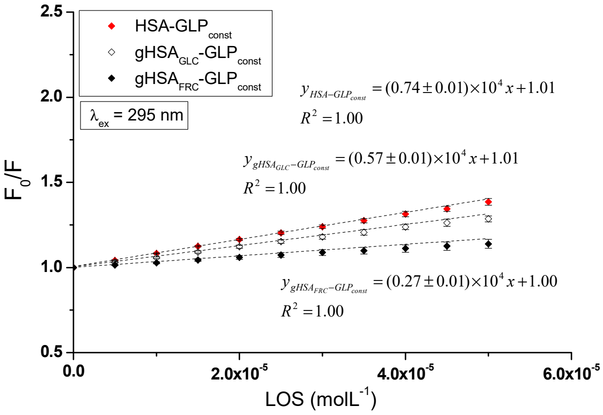

(**c**)

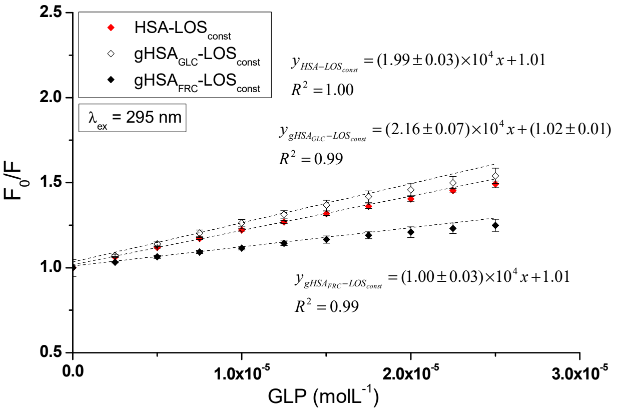

(**d**)

Figure S6. The Stern-Volmer curves for the binary (**a**) LOS–HSA, LOS–$gHSA_{GLC}$, LOS–$gHSA_{FRC}$; (**b**) GLP–HSA, GLP–$gHSA_{GLC}$, GLP–$gHSA_{FRC}$ and ternary systems (**c**) LOS–HSA–GLP_{const}, LOS–$gHSA_{GLC}$–GLP_{const}, LOS–$gHSA_{FRC}$–GLP_{const}; (**d**) GLP–HSA–LOS_{const}, GLP–$gHSA_{GLC}$–LOS_{const}, GLP–$gHSA_{FRC}$–LOS_{const}; λ_{ex} = 295 nm.

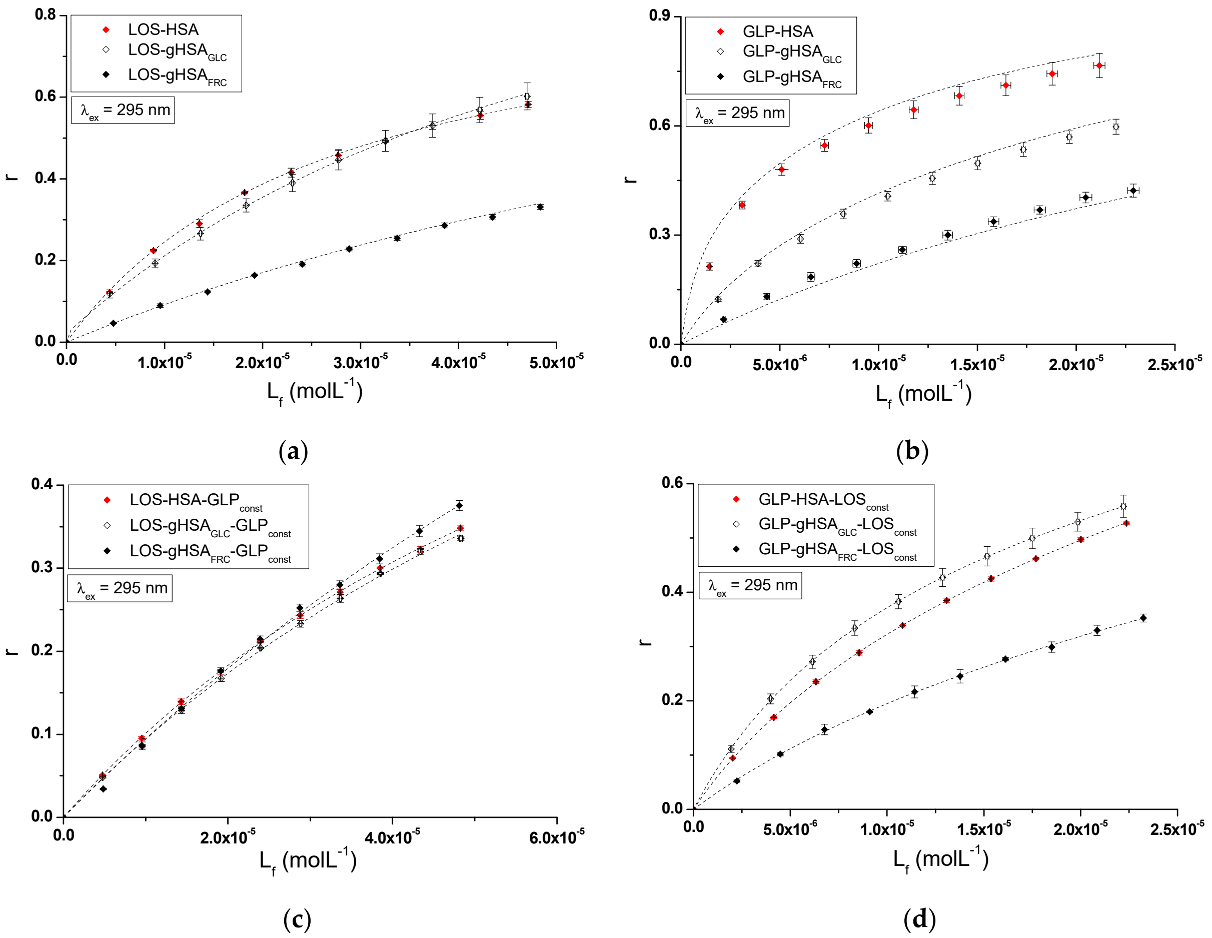

Figure S7. Binding isotherms of HSA, gHSA$_{GLC}$ and gHSA$_{FRC}$ at 5 × 10^{-6} mol·L^{-1} concentration with LOS at 5 × 10^{-6} mol·L^{-1} to 5 × 10^{-5} mol·L^{-1} and GLP at 2.5 × 10^{-6} mol·L^{-1} to 2.5 × 10^{-5} mol·L^{-1} concentrations in the binary (**a**) LOS–HSA, LOS–gHSA$_{GLC}$, LOS–gHSA$_{FRC}$; (**b**) GLP–HSA, GLP–gHSA$_{GLC}$, GLP–gHSA$_{FRC}$ and ternary systems (**c**) LOS–HSA–GLP$_{const}$, LOS–gHSA$_{GLC}$–GLP$_{const}$, LOS–gHSA$_{FRC}$–GLP$_{const}$; with GLP at 5 × 10^{-6} mol·L^{-1} concentration, (**d**) GLP–HSA–LOS$_{const}$, GLP–gHSA$_{GLC}$–LOS$_{const}$, GLP–gHSA$_{FRC}$–LOS$_{const}$ with LOS at 5 × 10^{-6} mol·L^{-1} concentration, λ_{ex} = 295 nm.

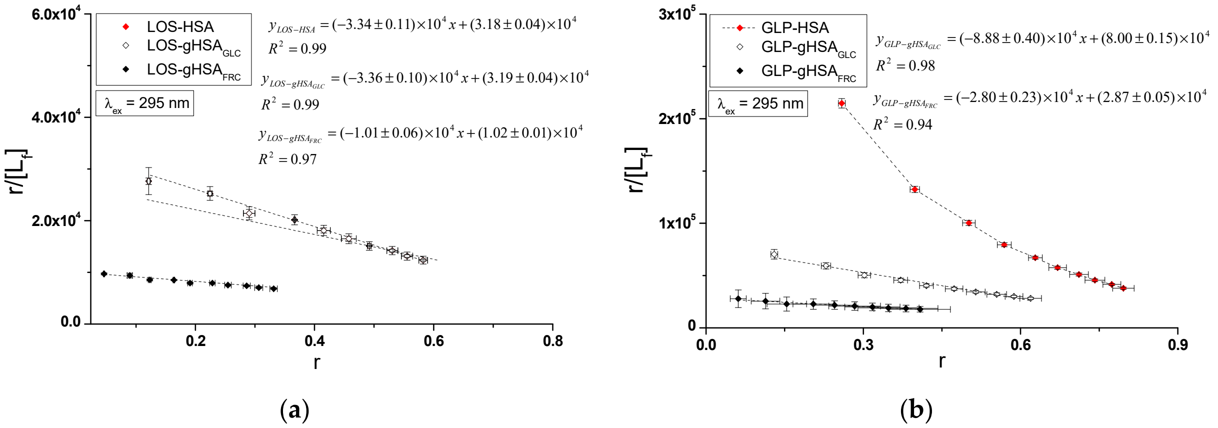

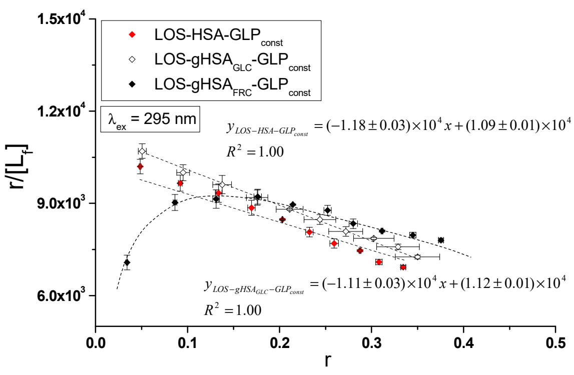

(**c**)

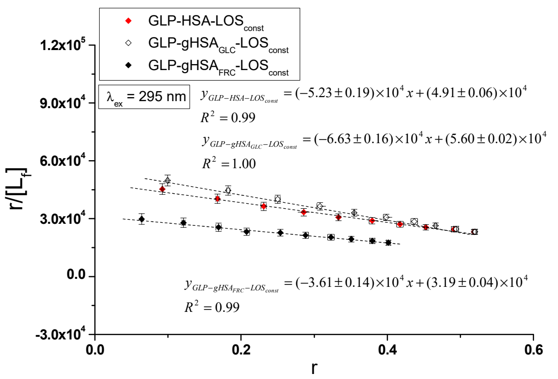

(**d**)

Figure S8. Scatchard plots for the binary (**a**) LOS–HSA, LOS–gHSA$_{GLC}$, LOS–gHSA$_{FRC}$; (**b**) GLP–HSA, GLP–gHSA$_{GLC}$, GLP–gHSA$_{FRC}$ and ternary systems (**c**) LOS–HSA–GLP$_{const}$, LOS–gHSA$_{GLC}$–GLP$_{const}$, LOS–gHSA$_{FRC}$–GLP$_{const}$; (**d**) GLP–HSA–LOS$_{const}$, GLP–gHSA$_{GLC}$–LOS$_{const}$, GLP–gHSA$_{FRC}$–LOS$_{const}$; λ_{ex} = 295 nm.

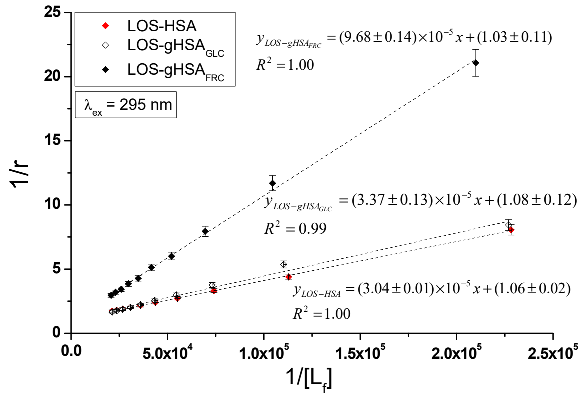

(**a**)

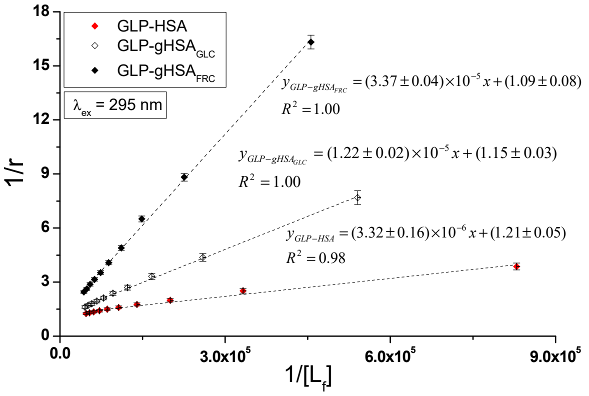

(**b**)

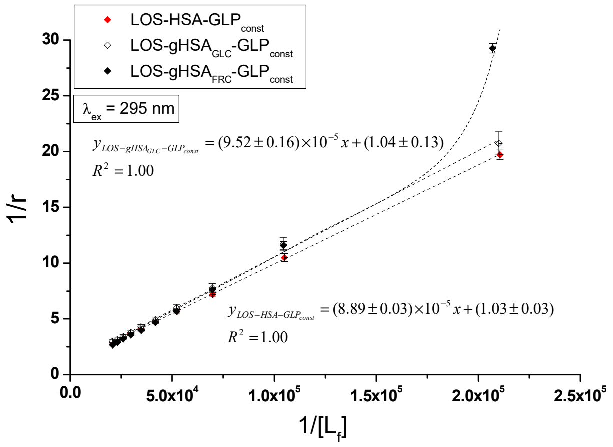

(**c**)

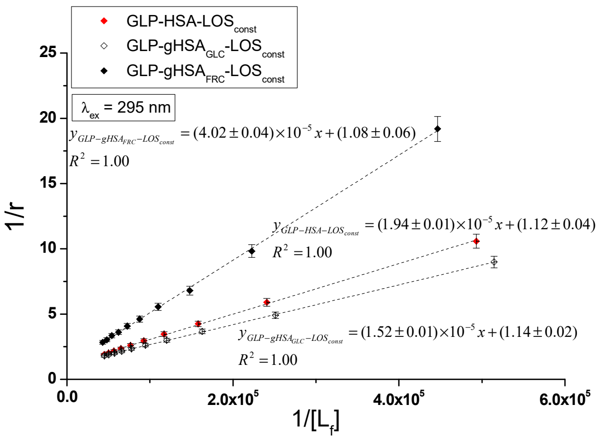

(**d**)

Figure S9. Klotz curves for the binary (**a**) LOS–HSA, LOS–gHSA$_{GLC}$, LOS–gHSA$_{FRC}$; (**b**) GLP–HSA, GLP–gHSA$_{GLC}$, GLP–gHSA$_{FRC}$ and ternary systems (**c**) LOS–HSA–GLP$_{const}$, LOS–gHSA$_{GLC}$–GLP$_{const}$, LOS–gHSA$_{FRC}$–GLP$_{const}$; (**d**) GLP–HSA–LOS$_{const}$, GLP–gHSA$_{GLC}$–LOS$_{const}$, GLP–gHSA$_{FRC}$–LOS$_{const}$; λ_{ex} = 295 nm.

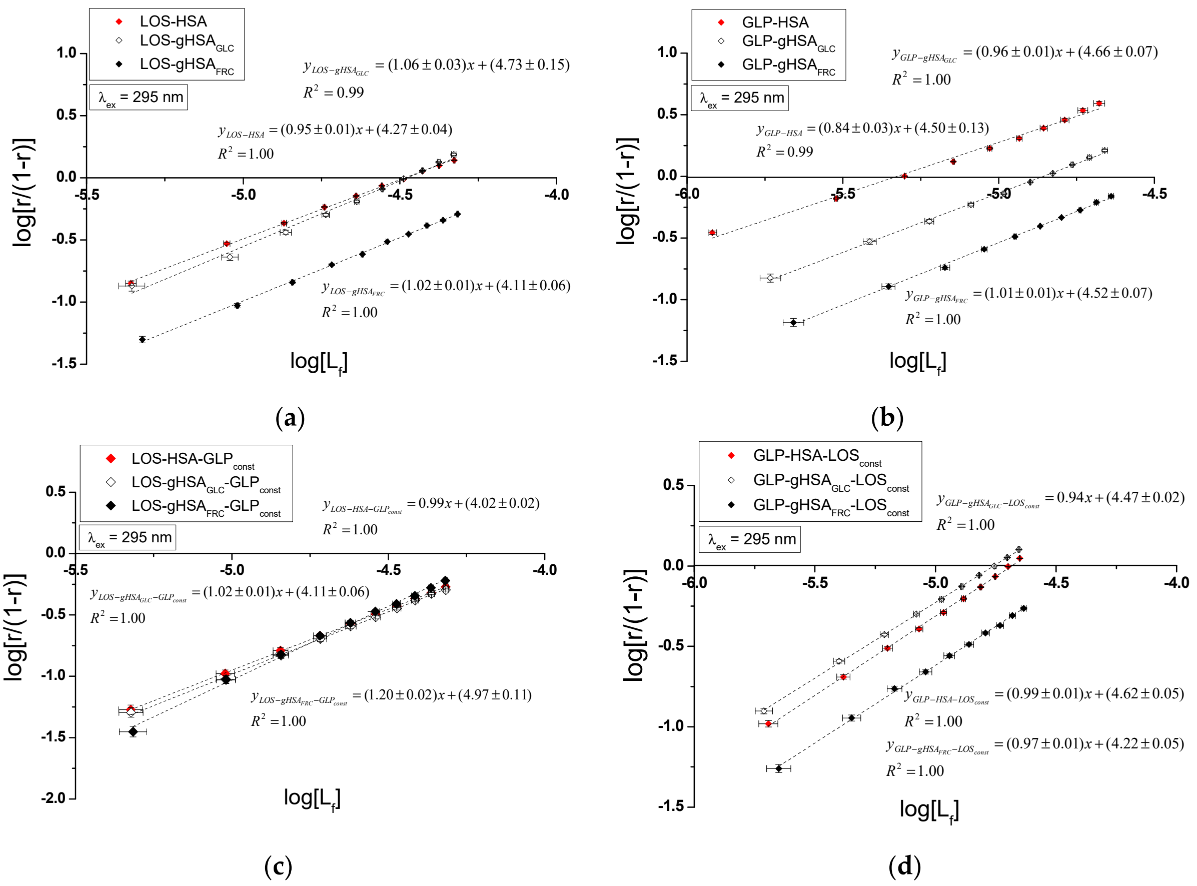

Figure S10. Hill plots for the binary (**a**) LOS–HSA, LOS–gHSA$_{GLC}$, LOS–gHSA$_{FRC}$; (**b**) GLP–HSA, GLP–gHSA$_{GLC}$, GLP–gHSA$_{FRC}$ and ternary systems (**c**) LOS–HSA–GLP$_{const}$, LOS–gHSA$_{GLC}$–GLP$_{const}$, LOS–gHSA$_{FRC}$–GLP$_{const}$; (**d**) GLP–HSA–LOS$_{const}$, GLP–gHSA$_{GLC}$–LOS$_{const}$, GLP–gHSA$_{FRC}$–LOS$_{const}$; λ_{ex} = 295 nm.
